# Stabilizing copper sites in coordination polymers toward efficient electrochemical C-C coupling

**DOI:** 10.1038/s41467-023-35993-4

**Published:** 2023-01-30

**Authors:** Yongxiang Liang, Jiankang Zhao, Yu Yang, Sung-Fu Hung, Jun Li, Shuzhen Zhang, Yong Zhao, An Zhang, Cheng Wang, Dominique Appadoo, Lei Zhang, Zhigang Geng, Fengwang Li, Jie Zeng

**Affiliations:** 1 Hefei National Research Center for Physical Sciences at the Microscale, CAS Key Laboratory of Strongly-Coupled Quantum Matter Physics, Key Laboratory of Surface and Interface Chemistry and Energy Catalysis of Anhui Higher Education Institutes, Department of Chemical Physics, University of Science and Technology of China, Hefei, Anhui 230026 P. R. China; 2 School of Chemical and Biomolecular Engineering and The University of Sydney Nano Institute, The University of Sydney, Sydney, NSW 2006 Australia; 3 Department of Applied Chemistry, National Yang Ming Chiao Tung University, Hsinchu, 300 Taiwan; 4 Frontiers Science Center for Transformative Molecules, Shanghai Jiao Tong University, Shanghai, 200240 China; 5 Australian Synchrotron, Clayton, VIC 3168 Australia

**Keywords:** Electrocatalysis, Electrocatalysis, Polymer synthesis, Sustainability

## Abstract

Electroreduction of carbon dioxide with renewable electricity holds promise for achieving net-zero carbon emissions. Single-site catalysts have been reported to catalyze carbon-carbon (C-C) coupling—the indispensable step for more valuable multi-carbon (C_2+_) products—but were proven to be transformed in situ to metallic agglomerations under working conditions. Here, we report a stable single-site copper coordination polymer (Cu(OH)BTA) with periodic neighboring coppers and it exhibits 1.5 times increase of C_2_H_4_ selectivity compared to its metallic counterpart at 500 mA cm^−**2**^. In-situ/operando X-ray absorption, Raman, and infrared spectroscopies reveal that the catalyst remains structurally stable and does not undergo a dynamic transformation during reaction. Electrochemical and kinetic isotope effect analyses together with computational calculations show that neighboring Cu in the polymer provides suitably-distanced dual sites that enable the energetically favorable formation of an *OCCHO intermediate post a rate-determining step of CO hydrogenation. Accommodation of this intermediate imposes little changes of conformational energy to the catalyst structure during the C-C coupling. We stably operate full-device CO_2_ electrolysis at an industry-relevant current of one ampere for 67 h in a membrane electrode assembly. The coordination polymers provide a perspective on designing molecularly stable, single-site catalysts for electrochemical CO_2_ conversion.

## Abstract (web summary)

Typically, Cu catalysts agglomerate under electrochemical conditions. Here, the authors report a coordination polymer catalyst with neighboring Cu sites which remain isolated and reduce CO_2_ to C_2_H_4_ with high selectivity and stability

## Introduction

Electrochemical CO_2_ reduction reaction (CO_2_RR) powered by renewable electricity is a promising approach to upcycle CO_2_ emissions in the form of commodity chemicals and fuels, helping store and transport intermittent renewables and add value to carbon capture and storage^1,2^. Ethylene (C_2_H_4_), a key precursor for the polymer industry, is one of the most valuable products of CO_2_RR^3–5^. Copper-based inorganic catalysts, because of their ability to promote carbon–carbon (C–C) coupling—the indispensable step for multi-carbon (C_2+_) products, have been extensively studied and optimized to improve CO_2_ to C_2_H_4_ conversion with industry-relevant current density, selectivity, and stability^6–8^.

Natural metalloenzymes catalyze CO_2_ activation (e.g., formate dehydrogenase, carbon monoxide dehydrogenase)^9,10^ and complicated C–C bond formation (e.g., in carbohydrate metabolism)^11,12^ at ambient conditions in a rapid, specific, and reversible manner. Materials scientists and synthetic inorganic chemists have long sought to emulate the enzymatic reactions through the biomimetic creation of structural and functional models of active sites. Most success has hitherto been seen in solution-phase molecular complexes, which have highly tunable ligand fields to mimic the coordinating environment of metals in enzymes^13–15^. Heterogenization of these functional complexes by immobilizing onto conductive substrates, anchoring the single metal sites into nitrogen-doped carbon supports, or embedding as secondary building units into porous solids has been demonstrated to improve the stability of the complexes and extend applications in solvents where the complexes have the difficulty of poor solubility^16,17^.

While advances have been achieved over these heterogenized molecular moieties—in single-site Cu form—for CO_2_RR to mono-carbon (C_1_) products such as carbon monoxide (CO) or methane (CH_4_)^18–20^, few examples have been shown for C_2+_ selectivity^21^. Nam et al. constructed a distorted Cu dimer in a metal-organic framework (MOF), HKUST-1, but found that the MOF was reduced to Cu clusters under CO_2_RR conditions and the C_2+_ selectivity came from the derived metallic Cu with low coordination numbers (CNs)^22^. A nitrogen-coordinated Cu single-site catalyst showed high selectivity toward ethanol in CO_2_ and CO electroreduction, while operando X-ray absorption spectroscopy (XAS) suggested that the isolated Cu sites were reversibly converted to Cu nanoparticles, serving as the true catalytically active species in operation^23,24^. It is in doubt whether single-site Cu—without deriving the initial solid-state or molecular materials to Cu agglomerations—can remain stable under CO_2_RR and catalyze C–C coupling.

C–C coupling involves the coupling of surface-bound *CO and/or *CHO intermediates, necessitating a suitable distance between at least two adjacent binding sites^25,26^. The large interatomic distances typically observed for single-site Cu catalysts would not allow for this process to take place, explaining in part why the heterogenized molecular moieties have to undergo agglomeration to catalyze C_2+_ production^27^. We posit that if we are able to construct neighboring Cu single sites that are sufficiently close and stable under working conditions, we would have the chance to enable C–C coupling on these isolated sites for C_2+_ products in CO_2_RR.

Here, we report a Cu-based quasi-one-dimensional (1D) coordination polymer catalyst that is stable under CO_2_RR conditions and catalyzes the CO_2_ to C_2_H_4_ conversion with high faradaic efficiency (FE) at industry-relevant current densities. The polymer, denoted as Cu(OH)BTA, is extended longitudinally through the coordination of Cu atoms with deprotonated 1,2,3-benzotriazole (1H-BTA) and bridged transversely via hydroxyl groups. Operando XAS as well as in-situ Raman and infrared spectroscopies suggest that Cu(OH)BTA remains structurally stable and does not undergo dynamic transformation during CO_2_RR. Electrochemical and kinetic isotope effect (KIE) analyses together with computational calculations rationalize the C–C coupling process: neighboring Cu atoms in the polymer provide suitably distanced dual Cu sites that enable the energetically favorable formation of an *OCCHO intermediate after a rate-determining step of CO hydrogenation. In a flow cell at 500 mA cm^−2^, the Cu(OH)BTA exhibits a total C_2+_ products FE of 73% and C_2_H_4_ FE of 57%, 1.3-time and 1.5-time, respectively, improvement compared to Cu control. Assembling the catalyst into a membrane electrode assembly (MEA) system enables stable full-device CO_2_ electrolysis at a total current of ~1 A for 67 h.

## Results and discussion

### Preparation and structure characterization

**Fig. 1:**
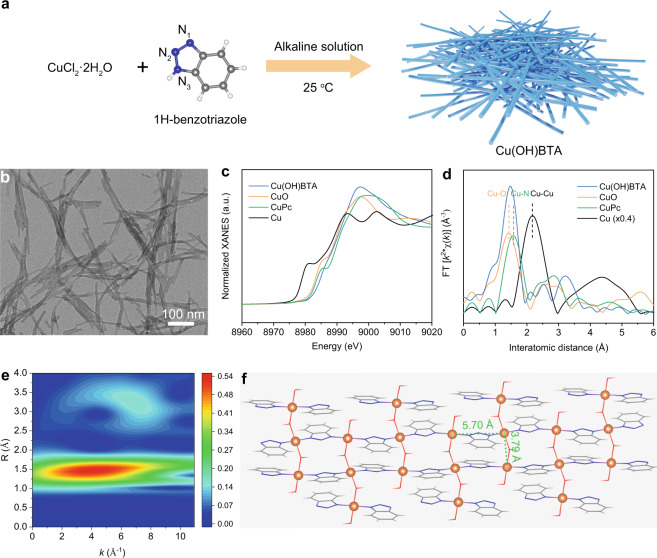
Preparation and structure characterization of Cu(OH)BTA. **a** Schematic illustration of the synthesis procedure. **b** TEM image of **Cu(OH)BTA**. Cu K-edge XANES spectrum (**c**) and EXAFS spectrum (**d**) of Cu(OH)BTA. The Cu K-edge XANES spectra of Cu foil, CuO, and copper(II) phthalocyanine (CuPc) were used as references. **e** Wavelet transforms (WT) for the *k*^2^-weighted Cu K-edge EXAFS signals of Cu(OH)BTA. **f** The wireframe model of Cu(OH)BTA. The isolated Cu atoms are highlighted in orange balls. The red, blue, gray, and white balls represent O, N, C, and H, respectively, in **a** and **f**.

We synthesized the quasi-1D Cu(OH)BTA through the reaction of ligand 1H-BTA and one equivalent of cupric chloride in an alkaline environment (Fig. 1a, see “Methods” for details). The Cu(OH)BTA has a uniform nanowire morphology with a width of 15–20 nm as shown in transmission electron microscopy (TEM, Fig. 1b). Scanning transmission electron microscopy energy-dispersive X-ray spectroscopy (STEM-EDS) mapping shows that N, Cu, and O disperse uniformly (Supplementary Fig. 1). The ratio of N and Cu elements is 3.1 (Supplementary Table 1). Isolate and dense Cu atoms were clearly observed via high-angle annular dark-field scanning transmission electron microscopy (Supplementary Fig. 2).

We conducted a series of spectral analyses to study the structure of Cu(OH)BTA. The N 1*s* X-ray photoelectron spectroscopic (XPS) spectrum of 1H-BTA was deconvoluted into two peaks located at 400.4 and 401.5 eV, which were attributed to pyridine-N and pyrrole-N, respectively (Supplementary Fig. 3)^28,29^. The binding energy of N in Cu(OH)BTA was slightly shifted to a lower, single-peak value of 399.8 eV. This suggested the electrons of N atoms were delocalized and the charge was distributed evenly over the three N atoms after coordinating with Cu^30,31^. The ratio of N and Cu calculated by XPS was 2.75 (Supplementary Table 2). The N_3_ in Cu(OH)BTA was deprotonated, revealed by the Fourier-transform infrared (FT-IR) spectroscopy (Supplementary Fig. 4). The solid-state ^13^C nuclear magnetic resonance (^13^C NMR) spectrum suggested that N_1_ and N_3_ of the triazole ring were coordinated with Cu to give a symmetrical structure of benzene in Cu(OH)BTA (Supplementary Fig. 5). Cu sites were bridged by hydroxyl group, evidenced by FT-IR spectroscopy and XPS (Supplementary Fig. 6)^32^. No Cl signal was detected in Cu(OH)BTA via XPS (Supplementary Fig. 7), suggesting that Cl^–^ did not involve in the coordination process.

We further conducted X-ray absorption fine structure (XAFS) spectroscopy to investigate the local environment of Cu in Cu(OH)BTA. As shown in Fig. 1c, the X-ray absorption near edge structure (XANES) spectrum of Cu(OH)BTA was close to that of CuO and copper phthalocyanine (CuPc), indicating a Cu(II) oxidation state in Cu(OH)BTA, consistent with Cu 2*p* XPS analysis (Supplementary Fig. 8)^33–35^. The extended X-ray absorption fine structure (EXAFS) spectra showed no Cu-Cu bond formed in Cu(OH)BTA (Fig. 1d). The peaks at 1.4 Å and 1.6 Å were attributed to Cu-O bond in CuO and Cu-N bond in CuPc, respectively. The wavelet transform of the Cu K-edge showed that both N and O were involved in the coordination with Cu (Fig. 1e and Supplementary Fig. 9).

Powder X-ray diffraction (PXRD) patterns showed an intense peak at 6.4°, a characteristic peak of macromolecular polymers (Supplementary Fig. 10)^36^. Pawley refinements of the PXRD patterns, in conjunction with density functional theory (DFT) calculations as well as spectroscopic analyses, suggested the structural model of Cu(OH)BTA in Fig. 1f. The Cu(OH)BTA extended longitudinally through repeated Cu-BTA coordination and bridged transversely via hydroxyl groups (Supplementary Fig. 11). The Cu-Cu distances were 5.70 Å along the longitudinal direction and 3.79 Å along the transverse direction. The calculated EXAFS using this structure showed a good fitting with that obtained experimentally, giving a CN of 2 for both Cu-N and Cu-O (Supplementary Fig. 12 and Table 3). The simulated structure of Cu(OH)BTA showed consistency in element composition and mass percentage with results of elemental analysis and inductively coupled plasma atomic emission spectroscopy (ICP-AES) (Supplementary Tables 4 and 5). Comparison of the difference in the ratio of N and Cu on the surface (via XPS) and in bulk (via elemental analysis and ICP-AES) results in a loss of surface BTA in the as-prepared Cu(OH)BTA to be 8.3%. Such BTA-defect Cu sites are likely to be active for CO_2_RR, evidenced in the following section.

### CO_2_RR performance

**Fig. 2:**
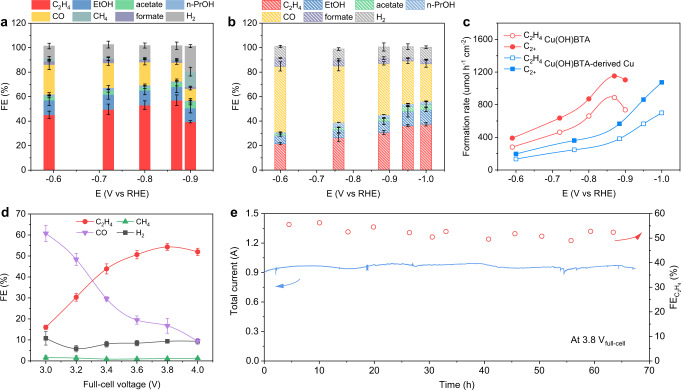
Performances of CO_2_RR over Cu(OH)BTA. Faradaic efficiencies (FEs) of all products under different applied potentials over Cu(OH)BTA (**a**) and Cu(OH)BTA-derived Cu (**b**) in 1 M KOH electrolyte. **c** The formation rates of C_2+_ products and C_2_H_4_ under different applied potentials over Cu(OH)BTA and Cu(OH)BTA-derived Cu in 1 M KOH electrolyte. **d** FEs of all gas products at the full-cell voltage range of 3.0–4.0 V measured in a membrane-electrode assembly (MEA) device. **e** C_2_H_4_ electrosynthesis in the MEA with a geometric electrode area of 4  cm^2^. The catalyst retains its total current and ethylene FE for 67 h. The error bars for FE uncertainty represent one standard deviation based on three independent samples.

We conducted CO_2_ electrolysis in a flow cell using 1 M KOH as the electrolyte (Supplementary Fig. 13). C_2_H_4_, CO, CH_4_, and H_2_ were identified and quantified as the gas products via gas chromatography. Ethanol (EtOH), acetate, and n-propanol (n-PrOH) were quantified by ^1^H NMR (see “Methods” and Supplementary Fig. 14 for details). FE_C2H4_ increased with applied potentials while FE_CO_ gradually decreased in the range from −0.59 V (versus reversible hydrogen electrode (RHE); All potentials are referenced to RHE unless otherwise noted) to −0.90 V (Fig. 2a). At −0.87 V, FE_C2H4_ reached its maxima of 57% with a partial current density of 285 mA cm^−2^ (Supplementary Fig. 15). The rest C_2+_ products included EtOH (11%), acetate (4%), and n-PrOH (1%), totaling a C_2+_ FE of 73%. More negative potentials resulted in a slight shift of selectivity toward CH_4_ and H_2_. Cu(OH)BTA showed a 1.5 times increase in C_2_H_4_ FE and 1.3 times increase in C_2+_ FE compared to the Cu(OH)BTA-derived Cu (preparation method vide infra), which only reached the highest C_2_H_4_ and C_2+_ FEs of 38% and 56%, respectively, at a more negative potential of −1.00 V (Fig. 2b). The Cu(OH)BTA demonstrated, at −0.87 V, a C_2_H_4_ half-cell energy efficiency (EE) of 31.3%, 1.6 times higher than that of the Cu(OH)BTA-derived Cu (Supplementary Table 6). The C_2+_ and C_2_H_4_ production rates over Cu(OH)BTA increased with applied potentials and reached, at −0.87 V, the maximum of 1151 and 887 μmol h^−1^ cm^−2^, respectively (Fig. 2c). ^13^CO_2_ isotope experiments showed the carbon in C_2+_ products was from the feed CO_2_ gas (Supplementary Fig. 16).

We further examined the full-cell CO_2_ electrolysis performance in a compact, commercialization-relevant MEA system (electrode geometric area = 4 cm^2^). Cu(OH)BTA catalysts were used as the cathode while iridium oxide supported on titanium mesh (IrO_2_/Ti) were used as the anode, and they were separated by an anion exchange membrane (Supplementary Figs. 17 and 18). The overall reaction is

1 $$2{{{\mbox{CO}}}}_{2}+2{{{\mbox{H}}}}_{2}{{\mbox{O}}}\to {{{\mbox{C}}}}_{2}{{{\mbox{H}}}}_{4}+3{{{\mbox{O}}}}_{2}{{{{{\rm{;}}}}}}\,{{E}}^{o}=1.15\,{{\mbox{V}}}$$

where *E*° is the equilibrium potential for the reaction at ambient conditions. The trend of C_2_H_4_ and CO selectivity at the full-cell voltage range of 3.0–4.0 V (without *i*R compensation) was similar to that obtained in the flow cell. A maxima FE of 54% for C_2_H_4_ was achieved at 3.8 V with a partial current density of 130 mA cm^−2^ (Fig. 2d and Supplementary Fig. 19). Initial stability test showed a stable operation of 67 h at a total current of 950 mA with an average FE of 52% for C_2_H_4_ (Fig. 2e). The Cu(OH)BTA catalyst outperformed start-of-the-art Cu-based catalysts that were derived from molecular compounds in FEs for C_2+_ and C_2_H_4_, current density, and durability (Supplementary Table 7). No noticeable change in morphology or coordination of the post-electrolysis Cu(OH)BTA sample was observed via SEM, high-resolution Cu XPS, and terahertz far-infrared spectroscopy (Supplementary Figs. 20–22).

### Active site under operating conditions

**Fig. 3:**
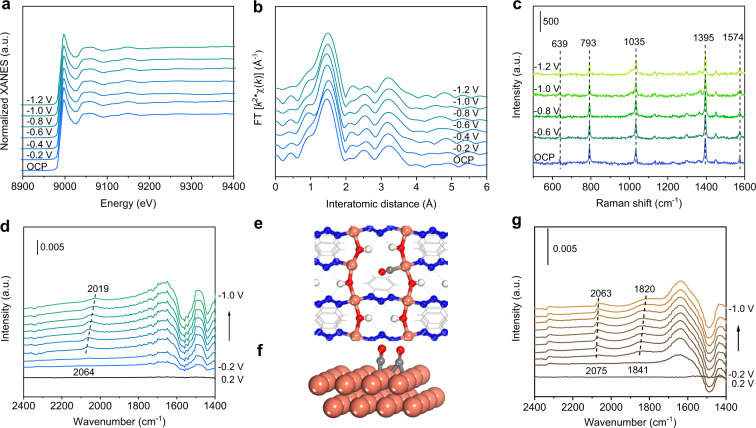
In-situ/Operando characterization of Cu(OH)BTA for CO_2_RR. Operando Cu K-edge XANES (**a**) and EXAFS (**b**) of Cu(OH)BTA at the applied potentials of −0.2 to −1.2 V under CO_2_ atmosphere. **c** In situ Raman spectra of Cu(OH)BTA at the applied potential of −0.6 to −1.2 V under CO_2_ atmosphere. The peaks at 639, 793, 1035, 1395, and 1574 cm^−1^ correspond to the vibrational modes of molecular structure in Cu(OH)BTA. **d** In situ ATR-SEIRAS spectra of Cu(OH)BTA in CO_2_-saturated 0.1 M KHCO_3_ electrolyte at the applied potential of −0.2 to −1.0 V. The background spectrum was taken at 0.2 V. DFT-predicted *CO intermediate configuration on Cu(OH)BTA (**e**) and Cu(111) slab (**f**). **g** In situ ATR-SEIRAS spectra of CO_2_RR on Cu(OH)BTA-derived Cu in CO_2_-saturated 0.1 M KHCO_3_ electrolyte. The background spectrum was taken at 0.2 V.

We interrogated catalytically active sites of Cu(OH)BTA using a suite of in-situ/operando spectroscopies. It has been recently shown that dynamic and reversible alterations take place in N-/O- chelated or covalent-organic-framework confined single-atom Cu and in immobilized CuPc^24,37^, demanding in-situ/operando techniques to identify the real active sites under operating conditions. We conducted operando XAFS spectroscopy of the Cu K-edge to explore the nature of Cu local environment in Cu(OH)BTA using a customized flow cell (Supplementary Fig. 23). The operando XANES spectra revealed an unchanged Cu oxidation state under CO_2_RR from −0.2 to −1.2 V (Fig. 3a). No Cu-Cu bond was discerned in the EXAFS (Fig. 3b), demonstrating that Cu remained as coordinated, atomically dispersed state under CO_2_RR condition.

To further verify the structural stability of Cu(OH)BTA under electroreduction conditions, we employed in situ vibrational spectroscopies to examine the catalyst and its adsorption behaviors of CO_2_RR intermediate(s). We did these additional verifications acknowledging that the lack of a metal–metal contribution in EXAFS does not necessarily indicate the lack of metallic clusters due to strong disorder or destructive interference of alternative contributions to total EXAFS signals^38^.

We first studied in-situ Raman spectroscopy of Cu(OH)BTA, which was conducted at an applied potential of −0.6 to −1.2 V during the CO_2_RR (Fig. 3c). At open-circuit potential (OCP), Cu(OH)BTA exhibited typical Raman shifts that correspond to the vibrational modes of molecular structure in Cu(OH)BTA (Supplementary Table 8). With increasing applied potentials, these peaks showed little change. This observation indicated high stability of the coordination structure of Cu(OH)BTA. Nevertheless, irreversible, partial decomposition of the Cu(OH)BTA or transient formation of BTA-stabilized Cu nanoclusters may also lead to similar observations.

We then performed in-situ FT-IR spectroscopy (Supplementary Fig. 24) to examine the above possibility. FT-IR is highly sensitive to the vibration mode of adsorbed species^39^. It is expected, for a certain CO_2_RR intermediate, only one mode should be observed because of the single-site nature of Cu in Cu(OH)BTA. As shown in Fig. 3d, one band in the range of 2064–2019 cm^−1^ appeared, which was attributed to electrogenerated *atop*-adsorbed CO (*CO_atop_) on Cu(OH)BTA. The redshift of band position with negative potentials is caused by the stark effect, confirming that the *CO was in-situ generated during CO_2_RR^40,41^. The CO was calculated to be adsorbed *atop* coordination-unsaturated Cu of Cu(OH)BTA (Fig. 3e), in agreement with the diffuse reflectance infrared Fourier transformations spectroscopy using CO as the probe molecule (Supplementary Fig. 25). By contrast, Cu(OH)BTA-derived Cu showed two bands: one at 2075–2063 cm^−1^ corresponds to asymmetric *CO_atop_ stretching vibration and another at 1841–1820 cm^−1^ associates with *bridge*-adsorbed CO (*CO_bridge_) (Fig. 3f, g)^42^. Prior reports have shown that the presence of *CO_bridge_, which necessitates at least two adjacent sites for its adsorption, is an indicator of the formation of Cu agglomerations^43,44^.

Collectively, the lack of the *CO_bridge_ in the in situ FT-IR spectroscopy, in conjunction with operando XAS and in situ Raman spectroscopic results, provides decisive evidence that the Cu(OH)BTA remains stable and does not undergo dynamic transformations during CO_2_RR.

### Origin of the stability and reaction mechanism under CO_2_RR conditions

To explore the stability of Cu(OH)BTA during CO_2_RR, we sought initially to carry out electrochemical CO reduction reaction (CORR) as it shares similar reaction mechanisms^45^. It is surprising to us that, after applying a constant current density of −100 mA cm^−2^ using 1 M KOH as the electrolyte, Cu(OH)BTA turned to metallic Cu within 10 min (Supplementary Fig. 26). Using Ar to replace CO resulted in similar decomposition of Cu(OH)BTA (Supplementary Figs. 27, 28, and Supplementary Table 9). The decomposition is irreversible and no residual BTA molecule was left on the resulting Cu surface (Supplementary Figs. 29 and 30). Cyclic voltammetry (CV) studies also showed instability of Cu(OH)BTA under Ar atmosphere within redox processes of Cu^2+^/Cu^1+^/Cu^0^ emerging only after 10 scans. By contrast, no redox process was observed under CO_2_ atmosphere in the same flow cell using 1 M KOH as electrolyte (Supplementary Figs. 31 and 32).

The instability of Cu(OH)BTA under both CORR and HER led us to posit that the strongly alkaline environment (i.e., elevated local pH >> 14) locally formed during either CORR or HER might deteriorate the stability of Cu(OH)BTA. This high local pH, by contrast, is not the case during CO_2_RR as CO_2_ reacts with OH^–^—locally generated or in bulk—to form carbonate and thus lowers local pH. In fact, prior modeling and experimental studies point to a pH lower than 14 in flow cells with 1 M KOH electrolyte^46,47^. To test this hypothesis, we carried out CO_2_RR using 10 M KOH as the electrolyte and found similar decomposition of Cu(OH)BTA (Supplementary Fig. 26). These results collectively suggest that the CO_2_RR creates a suitable local environment that prevents the Cu(OH)BTA from being exposed to harsh alkaline environment and thus keeps the molecular structure stable.

**Fig. 4:**
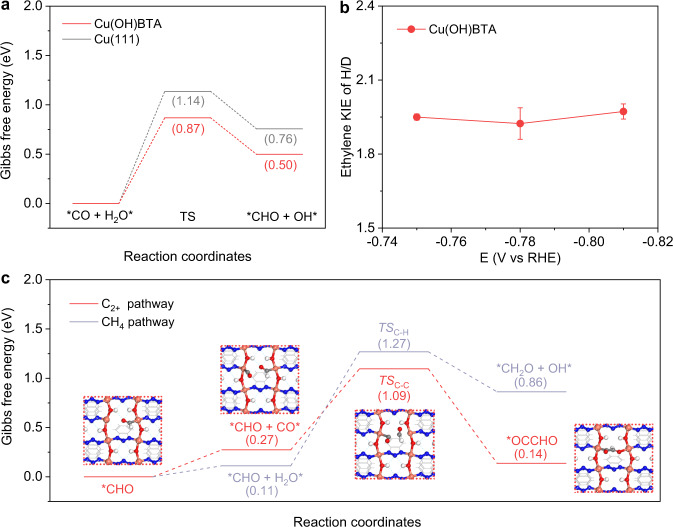
Investigation of Cu(OH)BTA stability under CO_2_RR. **a** Gibbs free energy diagram of hydrogenation of *CO on Cu(OH)BTA slab and Cu(111) slab. **b** KIE of H/D in CO_2_RR on Cu(OH)BTA under the potential range from −0.75 to −0.81 V. The error bars for ethylene KIE uncertainty represent one standard deviation based on three independent samples. **c** Gibbs free energy diagram of CO_2_RR to C_2+_ and CH_4_ pathway on Cu(OH)BTA slab. Inset figures: top-view geometries of intermediates (*CHO, *CHO + *CO, TS_C-C_, and *OCCHO) along CO_2_-to-C_2+_ pathway on Cu(OH)BTA slab.

We sought further to understand the reaction mechanism at a molecular level using DFT calculations. The optimized geometries showed that the neighboring two Cu sites with BTA defects have the most favorable energy profiles (Supplementary Fig. 33). The rate-determining step (RDS) along the CO_2_-to-C_2+_ path was calculated to be *CO hydrogenation by H_2_O to *CHO (Supplementary Figs. 34 and 35). This step on Cu(OH)BTA slab has an energy barrier of 0.87 V, which was 0.27 V lower than that on Cu(111) slab (Fig. 4a), in line with the experimentally observed higher C_2+_ selectivity over Cu(OH)BTA than that over Cu control. H/D KIE experiment using H_2_O and D_2_O as the electrolyte (i.e., proton source) showed a ratio of ethylene production rates (K_H_: K_D_) close to 2:1 (Fig. 4b), supporting a RDS involving the hydrogenation by H_2_O^48–50^. Post *CO hydrogenation, the *CHO favored energetically to couple with another *CO on the neighboring Cu site to form *OCCHO with a low energy barrier of 0.82 V for C–C coupling (Fig. 4c and Supplementary Figs. 36 and 37)^51,52^. Further hydrogenation of the *CHO by H_2_O, by contrast, encountered an unfavorable uphill energy barrier of 1.16 eV to form *OCH_2_, the key intermediate for the CH_4_ pathway (Supplementary Fig. 38).

We analyzed the distance of neighboring Cu sites during the adsorption of these key intermediates, namely *CO, *CHO, and *OCCHO and the values were in the range of 5.68–5.83 Å (Supplementary Fig. 39, a and b, Table 10). They were close to the Cu-Cu distance (5.70 Å) in the pristine Cu(OH)BTA structure. Such a similar distance minimizes conformational energy needs for structure reconstruction and thus imposes little damage to the molecular scaffold. The charge density difference plot of the C_2_ intermediate, *OCCHO, in Cu(OH)BTA slab showed that electrons, once accepted by Cu, tended to transfer to adsorbates instead of reducing the Cu sites (Supplementary Fig. 39c), avoiding Cu leaching from its coordination environment during CO_2_RR.

In summary, experimental and theoretical studies point to a Cu(OH)BTA coordination polymer with neighboring Cu sites that are stable during CO_2_RR to promote C–C coupling. The structural stability origins from a suitable local environment during CO_2_RR. The energetically favorable formation of *OCCHO intermediate requires little changes of conformational energy to accommodate it during C–C coupling. Our work provided a perspective on designing molecularly stable catalysts for CO_2_ activation and conversion to value-added chemicals.

## Methods

### Chemicals and materials

Copper(II) chloride dihydrate (CuCl_2_·2H_2_O, AR), copper (II) nitrate, trihydrate (Cu(NO_3_)_2_·3H_2_O, AR), potassium bicarbonate (KHCO_3_, AR), potassium hydroxide (KOH, AR), sodium hydroxide (NaOH, AR), ammonia (NH_3_·H_2_O, 25.0–28.0%), hydrochloric acid (HCl, CP, 36%–38%), isopropanol, methanol, and acetone were all purchased from Sinopharm Chemical Reagent Co. Ltd. (Shanghai, China). 1H-1,2,3-Benzotriazole (1H-BTA) (C_6_H_5_N_3_) was purchased from J&K Scientific. Copper(II) hydroxide (Cu(OH)_2_), copper(II) phthalocyanine (CuPc), 3-(trimethylsilyl)−1-propanesulfonic acid sodium salt (DSS), (dimethyl sulfoxide)-d6 (DMSO-d6), and Nafion solution (~5 wt.%) were purchased from Sigma-Aldrich. Proton exchange membrane (Nafion 115) was purchased from Dupont. Anion exchange membrane (Sustainion x37-50-grade-60) was purchased from Dioxide Materials. Titanium gauze (100 mesh) and iridium(III) chloride hydrate (IrCl_3_·3H_2_O) were obtained from Alfa Aesar. Carbon paper-based gas diffusion layers (GDLs, Sigracet 29 BC) were purchased from the Fuel Cell Store. Ultrapure Millipore water (resistivity 18.2 MΩ cm) was used for all experiments. All the chemicals were used without further purification.

### Synthesis of Cu(OH)BTA coordination polymer

0.85 g of CuCl_2_·2H_2_O was added into 50 mL of ultrapure water to obtain a blue aqueous solution. 0.60 g of 1H-BTA, 0.60 g of NaOH, and 100 mL of ultrapure water were mixed to obtain a colorless clear solution. The CuCl_2_ aqueous solution was quickly pulled into the mixture and then stirred at room temperature for 12 h. After that, the obtained blue colloid was centrifuged and washed with deionized H_2_O three times. Finally, the sediments were dried at 80 °C overnight under vacuum. The as-synthesized sample was used as the catalyst (denoted as Cu(OH)BTA) after grinding. Cu(OH)BTA-derived Cu was prepared by in-situ electroreduction at −200 mA cm^−2^ for 10 min under Ar atmosphere.

### Preparation of working electrodes

10 mg of the catalyst powder was dispersed in 2 mL of methanol by ultrasound for 30 min. 10 µL of Nafion solution (~5 wt.%) was added into the solution for another 30 min to get the catalyst ink. Then, the ink was air-brushed onto a piece of 2 cm × 4 cm GDL to prepare gas diffusion electrodes (GDEs). The loading amount was around 1.0 mg cm^−2^ which was determined by weighing the GDEs before and after air-brushing catalysts. The prepared electrodes were dried under an ambient environment for 12 h. This recipe was used to prepare electrodes used in operando spectroscopic characterization.

### Preparation of the anode IrO_2_/Ti mesh

The anode was prepared by a dip-coating and thermal decomposition method with some modification^53^. Typically, 30 mg of IrCl_3_·3H_2_O was dissolved in the mixture of 9 mL of isopropanol and 1 mL of 6 M HCl at 80 °C to prepare the dip-coating solution. A 2 cm × 2 cm Ti mesh was washed with water and acetone by ultrasound to remove oil stains, and then was etched in a 6 M HCl solution for 30 min. The etched Ti mesh was immersed into the above solution and then dried under infrared light. The dip-coat and drying processes were repeated several times. After that, the Ti mesh was calcinated in a furnace at 500 °C for 10 min. The IrO_2_/Ti electrode was prepared with a loading amount of around 2.0 mg cm^−2^.

### Electrochemical measurements

The CO_2_ electroreduction reaction was conducted in a flow cell or a membrane electrode assembly (MEA) system. The flow cell used a three-electrode system with an electrochemical workstation (CHI 1140c) as the potentiostat. In the flow cell, the prepared GDEs, Ag/AgCl electrode (3 M KCl), and Pt electrode were employed as the working, reference, and counter electrodes, respectively. The proton exchange membrane (Nafion 115) was used to conduct ions and separate the anode and cathode chambers. 1 M KOH was used as the electrolyte. 10 mL of catholyte was circulated in the cathode chamber at a constant rate of 5 mL min^−1^ by a peristaltic pump. Another 10 mL of anolyte was circulated in the anode chamber at the same rate. The gaseous 95% CO_2_ (5% N_2_ as internal standard) was flowed by the backside of the GDE at a rate of 20 standard cubic centimeters per minute (sccm), controlled by a mass flow meter. The geometric surface area of the catalyst was 1 cm^2^. The CO electroreduction reaction was conducted using the same experimental conditions except replacing CO_2_ with CO. All potentials in this work except those in the MEA experiments were measured against the Ag/AgCl electrode with *i*R compensation and were converted to the RHE scale based on the following equation:

2 $$E\,\left({{\mbox{vs. RHE}}}\right)=E\left({{\mbox{vs. Ag/AgCl}}}\right)+0.210+0.059\times {{{\mbox{pH}}}}-i{{\mbox{R}}}$$

All the electrocatalytic reactions were conducted at ambient pressure and temperature. The cell resistances (R) of 2 ohm in 1 M KOH electrolytes were measured by electrochemical impedance spectroscopy (EIS) under open circuit potentials (Supplementary Fig. 40).

The half-cell energy efficiency (EE) of ethylene in the flow cell was calculated by the following equation:

3 $${{{{{{\rm{EE}}}}}}}_{{{{{{\rm{half}}}}}}-{{{{{\rm{cell}}}}}}}=\frac{(1.23-{{{{{{\rm{E}}}}}}}_{{{{{{\rm{ethylene}}}}}}})\times {{{{{{\rm{FE}}}}}}}_{{{{{{\rm{ethylene}}}}}}}}{1.23-{{{{{{\rm{E}}}}}}}_{{{{{{\rm{applied}}}}}}}}$$

The overpotential of oxygen evolution is assumed to be zero. E_ethylene_ = 0.08 V (vs RHE) for CO_2_RR^54^.

The kinetic isotopic effect (KIE) of H/D over the catalysts was measured with a similar procedure except for replacing H_2_O with D_2_O.

The CV curves of the Cu(OH)BTA catalysts were recorded at a scan rate of 100 mV s^−1^ in the above flow cell. The used GDEs were collected and washed with water for further characterization.

For the MEA system, the anodic and cathodic flow fields were made of titanium and stainless steel, respectively. The exposure area of the two flow fields was both 4 cm^2^. The two-electrode model was used in the MEA. GDEs, anion exchange membrane, and IrO_2_/Ti mesh were assembled between two flow fields. A 3 mm thick PTFE gasket was placed between the membrane and anodic field to ensure the sealing of the system. 0.1 M KHCO_3_ anolyte was flowed through the anodic serpentine channel at a constant rate of 15 mL min^−1^ by a gas-liquid mix flow pump. The humidified feed gas (95% CO_2_ + 5% N_2_) was supplied to the cathodic serpentine channel at a constant rate of 20 sccm by a mass flow meter. For the stability test, the MEA was operated at a constant full-cell potential of 3.8 V. 10 L of 0.1 M KHCO_3_ anolyte was prepared to ensure that the composition of the electrolyte was nearly unchanged during the long electrolysis process. The gas products were analyzed at intervals of about ten hours.

### Quantification of products

The gaseous products were detected by online gas chromatography (GC2014, Shimadzu, Japan). The contained flame ionization detector (FID) was used to detect hydrocarbon products and the thermal conductivity detector (TCD) was used to detect H_2_, N_2,_ and CO. 5% N_2_ contained in feed gas was used as an internal standard to quantify the gas products. The liquid product was quantified by a 400 M Hz ^1^H-NMR spectrometer. Typically, 0.4 mL of the electrolyte after electrolysis was mixed with 0.1 mL DMSO-d6 and 0.1 mL of 6 mM DSS solution. DSS serves as an internal standard. The area ratio of the liquid product peaks to the DSS peak was compared to the standard curve to quantify the concentration of liquid products.

### Characterizations

The morphologies of samples were recorded by SEM (Zersss Supra 40) and TEM (Hitachi H-7650). HAADF-STEM images were taken on a field-emission transmission electron microscope (JEOL ARM-200F) operating at 200 kV accelerating voltage. The scanning transmission electron microscopy energy-dispersive X-ray spectroscopy (STEM-EDS) mapping images were taken on a high-resolution transmission electron microscopy (JEOL JEM-F200). X-ray diffraction patterns were recorded by using a Philips X’Pert Pro Super diffractometer with Cu-Kα radiation (λ = 1.54178 Å). The transmitted infrared spectra were carried out by a Fourier transform-infrared absorption (FT-IR) spectrometer (Thermo Scientific Nicolet is50). The Raman spectra were recorded by Lab RAM HR Evolution (Horiba). X-ray photoelectron spectroscopy (XPS) measurements were carried out on a VG ESCALAB MK II X-ray photoelectron spectrometer with an exciting source of Mg *K*_α_ = 1253.6 eV. The binding energies were corrected for specimen charging by referencing C 1*s* to 284.6 eV. The inductively coupled plasma atomic emission spectroscopy (ICP-AES) was carried out by Atom scan Advantage, Thermo Jarrell Ash, USA. The elemental analysis was recorded by Elementar vario EL cube. In-situ X-ray absorption spectroscopy (XAS) was performed in a home-made flow cell with a window sealed by Kapton tape. X-ray was allowed to transmit through the tape and GDEs. X-ray absorption near edge spectra (XANES) and extended X-ray absorption fine structure (EXAFS) were collected in the total-fluorescence-yield quick-scan mode at BL-44A beamline at National Synchrotron Radiation Research Center (NSRRC), Taiwan. Synchrotron terahertz far-infrared (THz-far IR) spectra were collected at the THz/far-IR beamline at the Australian Synchrotron with a Bruker IFS 125/HR Fourier Transform spectrometer at room temperature.

### XAFS analysis

XAFS data were processed with Demeter (v.0.9.26), in which an Athena software was employed to conduct energy calibration (using a Cu foil standard), spectral normalization, and linear combination fit (using the Cu K-edge XAFS spectra of Cu(OH)BTA and metallic Cu foil as standards). We then transformed the normalized EXAFS function, χ(E), in energy space to χ(*k*), where *k* is the photoelectron wave vector. χ(*k*) was multiplied by *k*^2^ to amplify the EXAFS oscillations in the mid-*k* region for assessing the interatomic interaction of Cu atoms. To differentiate the Cu K-edge EXAFS oscillation from different coordination shells, we applied Fourier transformation (from *k* space to R space) of the *k*^2^ -weighted χ(*k*) with a *k* range of 2.5-11 Å^−1^. Subsequently, EXAFS data recorded in R space was fitted using Artemis software in Demeter with FEFF6 program. The phase and amplitude functions of Cu-O, Cu-N, and Cu-Cu were calculated with FEFF. We then performed standard EXAFS fitting to extract structural parameters of Cu samples, including coordination number (CN) and bond distance (R).

### Computational methods

Periodic DFT calculations were conducted on Cu(OH)BTA coordination polymer and Cu(111) slab using the Vienna Ab Initio Simulation Package (VASP)^55^. The Perdew-Burke-Ernzerhof (PBE) functional and projector-augmented plane wave (PAW) were adopted to deal with exchange correlation and the ion-electron interaction^56,57^. The van der Waals interactions were described using the empirical DFT + D3 method^58^. A plane-wave basis set with a cutoff energy of 400 eV were selected for the calculations. All the atoms on Cu(OH)BTA monolayer were relaxed until the maximum force was <0.02 eV/Å. The electronic self-consistent field cycles were set at 10^−5^ eV. The zero-point energy corrections of key gas species were listed in Supplementary Table S11.

A monolayer model with 8 Cu, 8 OH, and 8 BTA ions is firstly cut from vdW-stacking Cu(OH)BTA bulk phase. As the preparation environment is rich of OH, it is reasonably supposed that BTA vacancies exist on the top layer. Therefore, one BTA vacancy is created so as to expose under-coordinated Cu sites. A 4 × 4 Cu(111) supercell with 64 atoms is established for comparison. The vacuum space along the Z direction was larger than 10 Å to avoid the interactions between period images. The Brillouin zone was sampled by a 2 × 2 × 1 k points mesh for monolayer slab and 3 × 3 × 1 for Cu(111) slab.

The Gibbs free energy change is defined as ΔG = ΔE + ΔZPE−TΔS, with ΔE obtained from DFT calculations, ΔZPE indicating the corrections in zero-point energies, and ΔS the change of entropy. It is assumed that S = 0 for all the adsorbed species. According to the model of computational hydrogen electrode (CHE), at U = O, the free energy change of proton-coupled electron transfer is equivalent to hydrogen production, namely H^+^ + e^−^ = 1/2 H_2_^59^. The adsorption energy of *OCCHO species is thus defined as Eads = E_*OCCHO_ + slab – E_slab_ – 2 E_*CO_ – 0.5 E_*H2_. The charge transfer between the *OCCHO species and Cu(OH)BTA species was analyzed using the Bader charge.

### In situ Raman measurements

In-situ Raman was carried out using Lab RAM HR Evolution (Horiba) in a customized flow cell with a 532 nm laser with 10% intensity. 0.1 M KOH electrolyte was used to reduce the interference of fluorescence effect to the Raman spectra. Each spectrum was collected by integration twice, 60 s per integration. The applied potentials ranged from −0.6 to −1.2 V vs. RHE and the in-situ spectra were collected at 0.2 V intervals. One sets of spectra were collected under CO_2_ with a flow rate of 20 sccm.

### In-situ FTIR measurements

In-situ FTIR was carried out using Thermo Scientific Nicolet iS50 FTIR spectrometer with internal reflection configuration at room temperature. An Au film was first deposited on a smooth surface of a Si prism. The air-brush method was used to completely cover the Au film by the catalysts. CO_2_-saturated 0.1 M KHCO_3_ was used in the measurements. All spectra were collected in absorbance at a resolution of 4 cm^−1^ while linear sweep voltammetry was conducted at the same time with a scan rate of 4 mV s^−1^.

### DRIFTs measurements

DRIFTs measurements were conducted in an elevated-pressure cell (DiffusIR Accessory PN 041-10XX) using Thermo Scientific Nicolet iS50 FTIR spectrometer with a wavenumber resolution of 4 cm^−1^ at room temperature. Background spectra were obtained after flowing under 1 bar of Ar with the rate of 30 sccm at 110 °C for 1 h, following by cooling to 25 °C. The DRIFTs spectra were obtained after purging by 1 bar of CO with the rate of 20 sccm at 25 °C for 30 min, then purging by 1 bar of Ar with the rate of 30 sccm at 25 °C for 1 h.

## Supplementary information

Supplementary Information

## Data Availability

All the data that support the findings of this study are available from the corresponding author on reasonable request. Source data are provided with this paper.

## Source data

Source Data

## References

[1] Chu S, Cui Y, Liu N (2017). The path towards sustainable energy. Nat. Mater..

[2] Zhu P, Wang H (2021). High-purity and high-concentration liquid fuels through CO_2_ electroreduction. Nat. Catal..

[3] Dinh C-T (2018). CO_2_ electroreduction to ethylene via hydroxide-mediated copper catalysis at an abrupt interface. Science.

[4] Zhu Q (2019). Carbon dioxide electroreduction to C_2_ products over copper-cuprous oxide derived from electrosynthesized copper complex. Nat. Commun..

[5] Huang D-S (2022). A stable and low-cost metal-azolate framework with cyclic tricopper active sites for highly selective CO_2_ electroreduction to C_2+_ products. ACS Catal..

[6] Zhong D (2020). Coupling of Cu(100) and (110) facets promotes carbon dioxide conversion to hydrocarbons and alcohols. Angew. Chem. Int. Ed..

[7] Lyu Z (2021). Controlling the surface oxidation of Cu nanowires improves their catalytic selectivity and stability toward C_2+_ products in CO_2_ reduction. Angew. Chem. Int. Ed..

[8] Li H (2021). High-rate CO_2_ electroreduction to C_2+_ products over a copper-copper iodide catalyst. Angew. Chem. Int. Ed..

[9] Miller M (2019). Interfacing formate dehydrogenase with metal oxides for the reversible electrocatalysis and solar-driven reduction of carbon dioxide. Angew. Chem. Int. Ed..

[10] Reginald SS, Etzerodt M, Fapyane D, Chang IS (2021). Functional expression of a Mo–Cu-dependent carbon monoxide dehydrogenase (CODH) and its use as a dissolved CO bio-microsensor. ACS Sens..

[11] Breuer M, Hauer B (2003). Carbon-carbon coupling in biotransformation. Curr. Opin. Biotechnol..

[12] Miao Y, Rahimi M, Geertsema EM, Poelarends GJ (2015). Recent developments in enzyme promiscuity for carbon-carbon bond-forming reactions. Curr. Opin. Chem. Biol..

[13] Wu W, Huang L, Wang E, Dong S (2020). Atomic engineering of single-atom nanozymes for enzyme-like catalysis. Chem. Sci..

[14] Lv B (2021). Controlling oxygen reduction selectivity through steric effects: electrocatalytic two-electron and four-electron oxygen reduction with cobalt porphyrin atropisomers. Angew. Chem. Int. Ed..

[15] Cheplakova AM (2019). Structural diversity of zinc(ii) coordination polymers with octafluorobiphenyl-4,4’-dicarboxylate based on mononuclear, paddle wheel and cuboidal units. CrystEngComm.

[16] Rooney CL, Wu Y, Tao Z, Wang H (2021). Electrochemical reductive N-methylation with CO_2_ enabled by a molecular catalyst. J. Am. Chem. Soc..

[17] Franco F, Rettenmaier C, Jeon HS, Cuenya BR (2020). Transition metal-based catalysts for the electrochemical CO_2_ reduction: from atoms and molecules to nanostructured materials. Chem. Soc. Rev..

[18] Wang Y-R (2021). Implanting numerous hydrogen-bonding networks in Cu-porphyrin-based nanosheet to boost CH_4_ selectivity in neutral media CO_2_ electroreduction. Angew. Chem. Int. Ed..

[19] Zhang X (2020). Molecular engineering of dispersed nickel phthalocyanines on carbon nanotubes for selective CO_2_ reduction. Nat. Energy.

[20] Nguyen TN, Salehi M, Le QV, Seifitokaldani A, Dinh CT (2020). Fundamentals of electrochemical CO_2_ reduction on single-metal-atom catalysts. ACS Catal..

[21] Bao H (2021). Isolated copper single sites for high-performance electroreduction of carbon monoxide to multicarbon products. Nat. Commun..

[22] Nam D-H (2018). Metal-organic frameworks mediate Cu coordination for selective CO_2_ electroreduction. J. Am. Chem. Soc..

[23] Fontecave M (2019). Electroreduction of CO_2_ on single-site copper-nitrogen-doped carbon material: selective formation of ethanol and reversible restructuration of the metal sites. Angew. Chem. Int. Ed..

[24] Xu H (2020). Highly selective electrocatalytic CO_2_ reduction to ethanol by metallic clusters dynamically formed from atomically dispersed copper. Nat. Energy.

[25] Qiu X-F (2022). A stable and conductive covalent organic framework with isolated active sites for highly selective electroreduction of carbon dioxide to acetate. Angew. Chem. Int. Ed..

[26] Sha Y (2022). Anchoring ionic liquid in copper electrocatalyst for improving CO_2_ conversion to ethylene. Angew. Chem. Int. Ed..

[27] Guan A (2020). Boosting CO_2_ electroreduction to CH_4_ via tuning neighboring single-copper sites. ACS Energy Lett..

[28] Artyushkova K (2013). Density functional theory calculations of XPS binding energy shift for nitrogen-containing graphene-like structures. Chem. Commun..

[29] Liu Y (2020). A highly efficient metal-free electrocatalyst of F-doped porous carbon toward N_2_ electroreduction. Adv. Mater..

[30] Mirarco A, Francisa SM, Baddeley CJ, Glisentib A, Grillo F (2018). Effect of the pH in the growth of benzotriazole model layers at realistic environmental conditions. Corros. Sci..

[31] Hashemi T, Hogarth CA (1988). The mechanism of corrosion inhibition of copper in NaCl solution by benzotriazole studied by electron spectroscopy. Electrochim. Acta.

[32] Nguyen DLT (2017). Selective CO_2_ reduction on zinc electrocatalyst: the effect of zinc oxidation state induced by pretreatment environment. ACS Sustain. Chem. Eng..

[33] Hinokuma S, Kawabata Y, Kiritoshi S, Matsuki S, Machida M (2017). Operando XAFS studies of supported copper oxides for catalytic ammonia combustion. J. Ceram. Soc. Jpn..

[34] Wang Y, Niu C, Zhu Y, He D, Huang W (2020). Tunable syngas formation from electrochemical CO_2_ reduction on copper nanowire arrays. ACS Appl. Energy Mater..

[35] Xu H (2020). Cation exchange strategy to single-atom noble-metal doped CuO nanowire arrays with ultralow overpotential for H_2_O splitting. Nano Lett..

[36] Wang R (2021). Partial coordination-perturbed Bi-Copper sites for selective electroreduction of CO_2_ to hydrocarbons. Angew. Chem. Int. Ed..

[37] Xu Y (2021). Low coordination number copper catalysts for electrochemical CO_2_ methanation in a membrane electrode assembly. Nat. Commun..

[38] Wang M, Árnadóttir L, Xu ZJ, Feng Z (2019). In situ X-ray absorption spectroscopy studies of nanoscale electrocatalysts. Nano-Micro Lett..

[39] Li X, Yang X, Zhang J, Huang Y, Liu B (2019). In situ/operando techniques for characterization of single-atom catalysts. ACS Catal..

[40] Wei X (2020). Highly selective reduction of CO_2_ to C_2+_ hydrocarbons at copper/polyaniline interfaces. ACS Catal..

[41] Gunathunge CM, Ovalle VJ, Li Y, Janik MJ, Waegele MM (2018). Existence of an electrochemically inert CO population on Cu electrodes in alkaline pH. ACS Catal..

[42] Chou T-C (2020). Controlling the oxidation state of the Cu electrode and reaction intermediates for electrochemical CO_2_ reduction to ethylene. J. Am. Chem. Soc..

[43] Shen H (2022). Asymmetrical C-C coupling for electroreduction of CO on bimetallic Cu-Pd catalysts. ACS Catal..

[44] Sun H (2021). Promoting ethylene production over a wide potential window on Cu crystallites induced and stabilized via current shock and charge delocalization. Nat. Commun..

[45] Todorova TK, Schreiber MW, Fontecave M (2020). Mechanistic understanding of CO_2_ reduction reaction (CO_2_RR) toward multicarbon products by heterogeneous copper-based catalysts. ACS Catal..

[46] Chen X (2020). Electrochemical CO_2_-to-ethylene conversion on polyamine-incorporated Cu electrodes. Nat. Catal..

[47] Lu X (2020). In situ observation of the pH gradient near the gas diffusion electrode of CO_2_ reduction in alkaline electrolyte. J. Am. Chem. Soc..

[48] Ma W (2020). Electrocatalytic reduction of CO_2_ to ethylene and ethanol through hydrogen-assisted C-C coupling over fluorine-modified copper. Nat. Catal..

[49] Wuttig A, Yaguchi M, Motobayashi K, Osawa M, Surendranath Y (2016). Inhibited proton transfer enhances Au-catalyzed CO_2_-to-fuels selectivity. Proc. Natl Acad. Sci. USA.

[50] Liu P (2016). Photochemical route for synthesizing atomically dispersed palladium catalysts. Science.

[51] Zhu H-L (2022). A porous π–π stacking framework with dicopper(I) sites and adjacent proton relays for electroreduction of CO_2_ to C_2+_ products. J. Am. Chem. Soc..

[52] Zhao Z-H, Zhu H-L, Huang J-R, Liao P-Q, Chen X-M (2022). Polydopamine coating of a metal-organic framework with Bi-copper sites for highly selective electroreduction of CO_2_ to C_2+_ products. ACS Catal..

[53] Gabardo CM (2019). Continuous carbon dioxide electroreduction to concentrated multi-carbon products using a membrane electrode assembly. Joule.

[54] Kuhl KP, Cave ER, Abram DN, Jaramillo TF (2012). New insights into the electrochemical reduction of carbon dioxide on metallic copper surfaces. Energy Environ. Sci..

[55] Kressea G, Furthmüller J (1996). Efficiency of ab-initio total energy calculations for metals and semiconductors using a plane-wave basis set. Comput. Mater. Sci..

[56] Kresse G, Joubert D (1999). From ultrasoft pseudopotentials to the projector augmented-wave method. Phys. Rev. B.

[57] Perdew JP, Burke K, Ernzerhof M (1996). Generalized gradient approximation made simple. Phys. Rev. Lett..

[58] Grimme S, Ehrlich S, Goerigk L (2011). Effect of the damping function in dispersion corrected density functional theory. J. Comput. Chem..

[59] Peterson AA, Abild-Pedersen F, Studt F, Rossmeisl J, Nørskov JK (2010). How copper catalyzes the electroreduction of carbon dioxide into hydrocarbon fuels. Energy Environ. Sci..

