# Supplementary material for: Stabilizing copper sites in coordination polymers toward efficient electrochemical C-C coupling
Source: Nat Commun. 2023 Jan 30;14:474. doi: 10.1038/s41467-023-35993-4 (PMC9884666; doi:10.1038/s41467-023-35993-4)
Supplement: Supplementary file 1 — Supplementary Information [file 41467_2023_35993_MOESM1_ESM.pdf]

Supplementary information for

Stabilizing copper sites in coordination polymers toward efficient electrochemical C-C coupling

Liang et al.

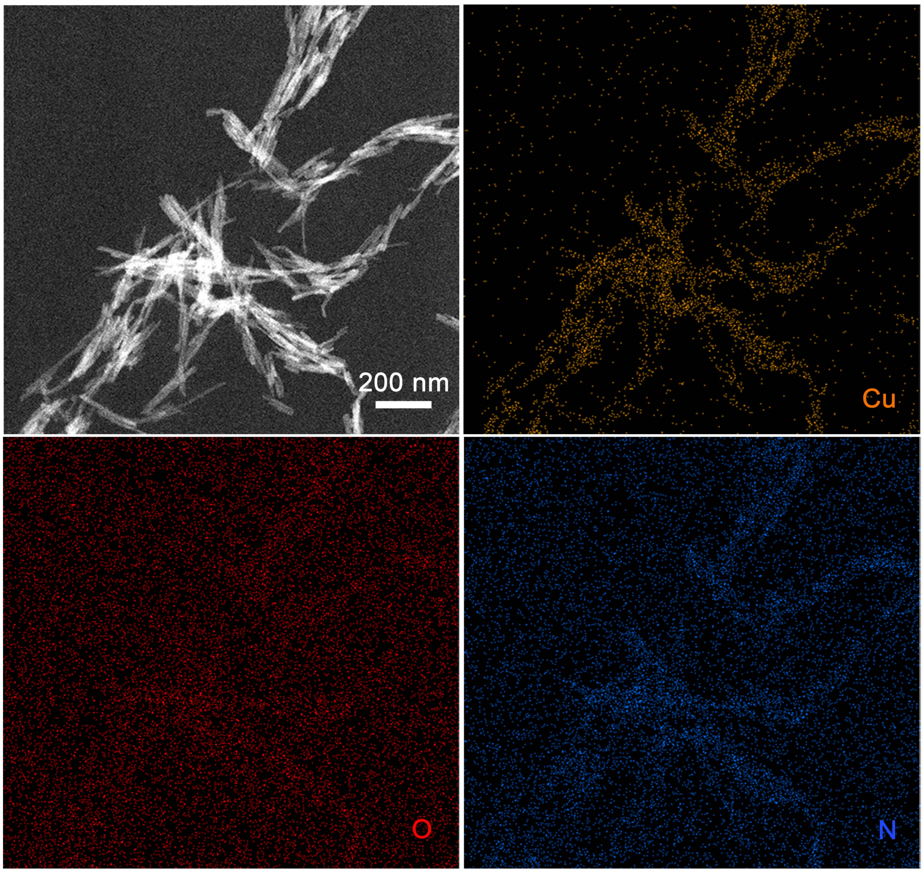

Supplementary Fig. 1 | Scanning transmission electron microscopy energy-dispersive X-ray spectroscopy (STEM-EDS) mapping of Cu(OH)BTA, showing the uniform dispersion of Cu, N, and O in the coordination polymer.

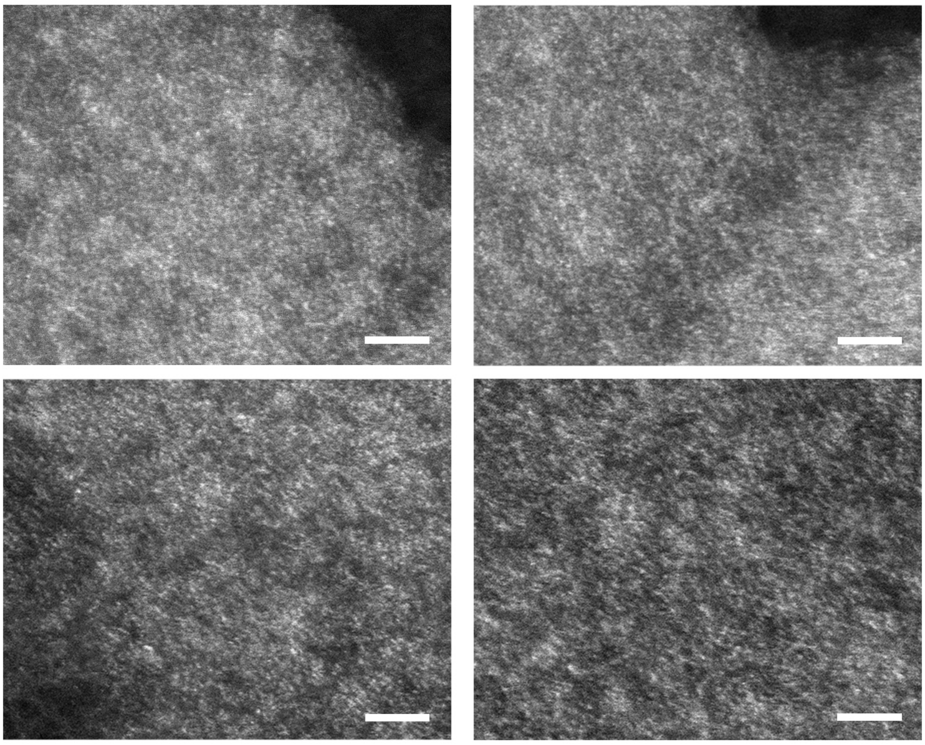

Supplementary Fig. 2 | High-angle annular dark-field scanning transmission electron microscopy (HAADF-STEM) image of Cu(OH)BTA in four different areas. The white dots are isolated and dense Cu atoms. The scale bars in the HAADF-STEM images are 2 nm.

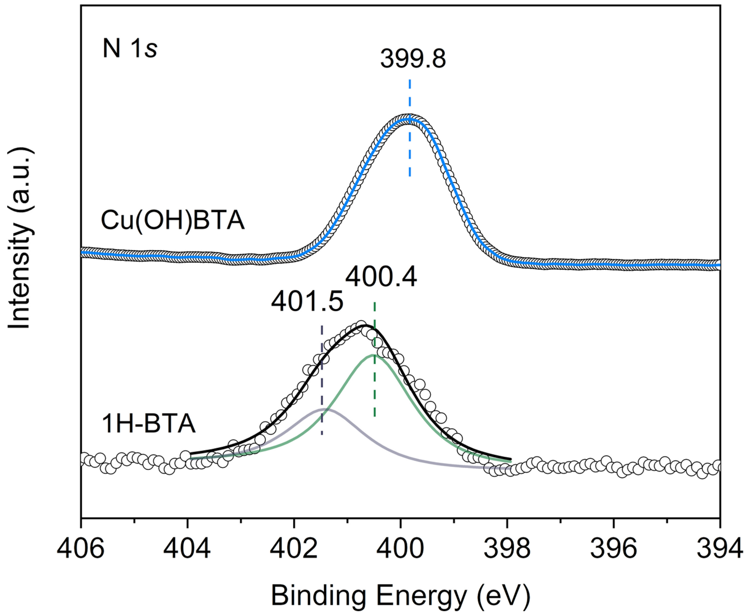

Supplementary Fig. 3 | N 1*s* XPS spectra of 1,2,3-benzotriazole (1H-BTA) molecule and Cu(OH)BTA.

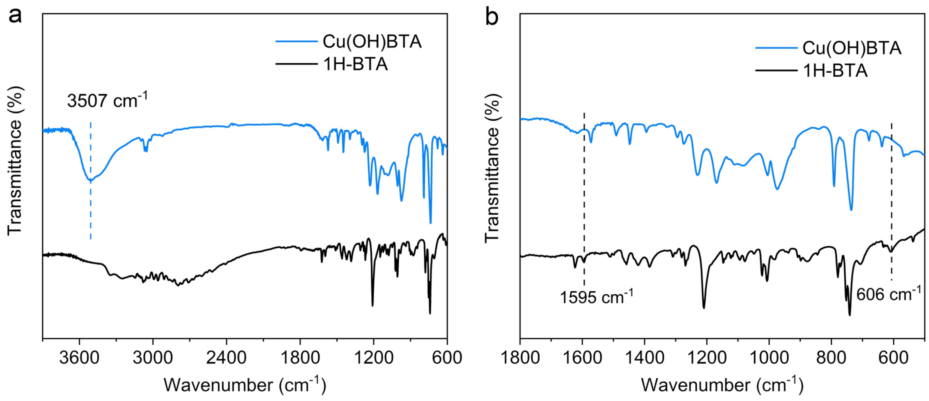

Supplementary Fig. 4 | Fourier-transform infrared spectroscopy (FT-IR). The full spectra (a) and magnified spectra of 1H-BTA and Cu(OH)BTA (b). The wide N-H stretching (from 3300 to 2600 cm^{-1}) is absent in Cu(OH)BTA. The peak at 3507 cm^{-1} in Cu(OH)BTA is attributed to the hydroxyl group.[1] No peak at 1600 cm^{-1} suggests that the hydroxyl comes from the structure of Cu(OH)BTA rather than water molecules. The absence of N-H in-plane bending (~1595 cm^{-1}) and N-H out-of-plane bending (~606 cm^{-1}) in Cu(OH)BTA indicates that the N atom in 1H-BTA is deprotonated.[2]

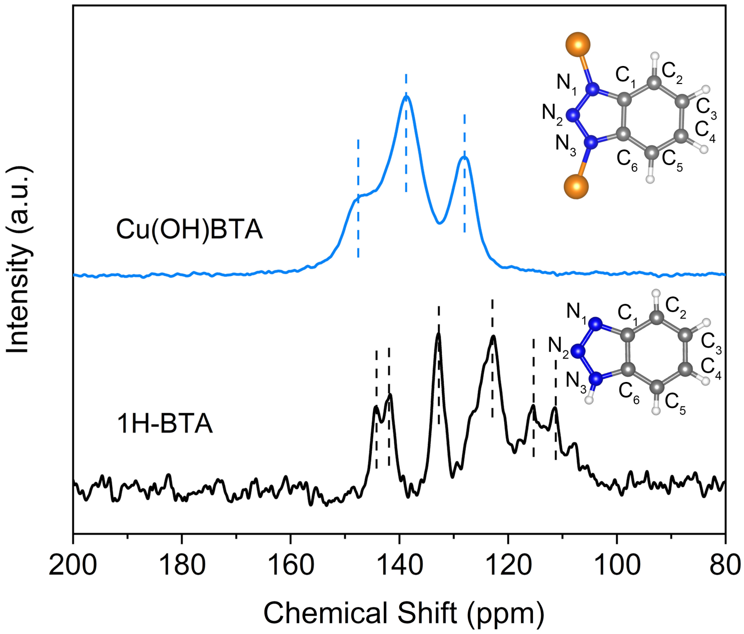

Supplementary Fig. 5 | ^{13}C solid-state NMR spectra of 1H-BTA molecule and Cu(OH)BTA. Inset: corresponding 1H-BTA structure before and after coordination with Cu. The ^{13}C solid-NMR of 1H-BTA showed six peaks at 144 ppm, 141 ppm, 132 ppm, 122.8 ppm, 115.4 ppm, 111.4 ppm, which were corresponding to six carbon atoms in the benzene ring of 1H-BTA. In Cu(OH)BTA, three peaks at 147 ppm, 138.8 ppm, and 128.2 ppm were attributed to ^{13}C signals of all carbon atoms, suggesting three types of chemical environment of carbon atoms. Symmetrical molecular structures were formed when 1H-BTA was coordinated with Cu.[3] Combined with the elemental analysis, Cu was determined to be coordinated with N_1 and N_3.

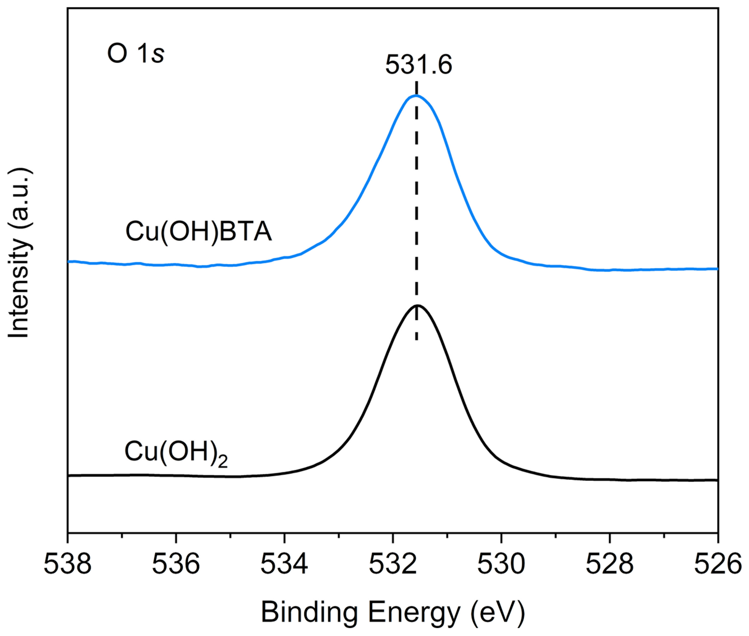

Supplementary Fig. 6 | O 1*s* XPS of Cu(OH)BTA. Cu(OH)BTA showed the same O 1*s* binding energy as that in the reference $Cu(OH)_2$ sample.

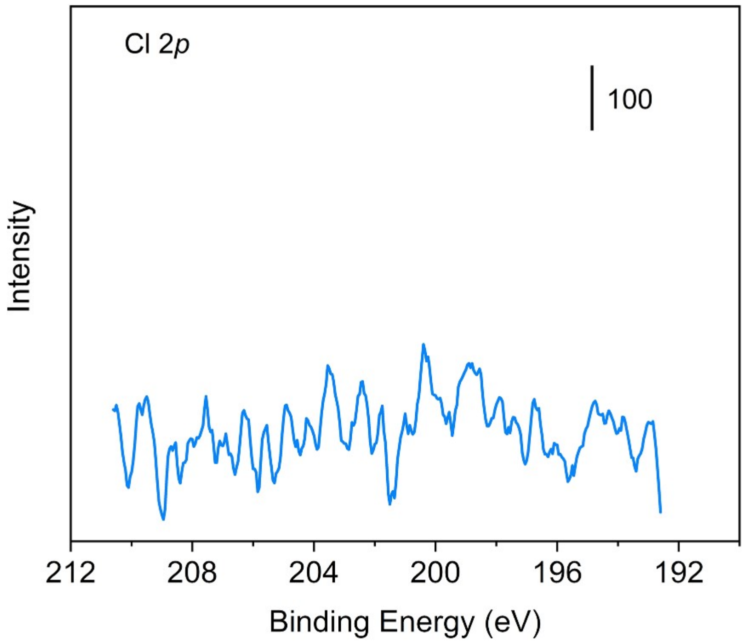

Supplementary Fig. 7 | Cl 2*p* XPS of Cu(OH)BTA. The absence of Cl 2*p* signal illustrated that the chloride ions in the $CuCl_2$ precursor were completely replaced and did not involve coordination in Cu(OH)BTA.

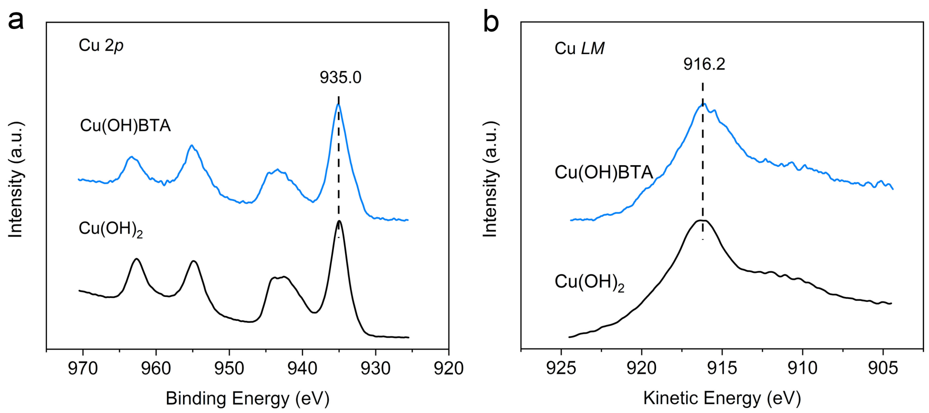

Supplementary Fig. 8 | High-resolution Cu XPS. Cu 2*p* spectra (a) and Cu *LM* spectra (b) of Cu(OH)BTA and $Cu(OH)_2$.

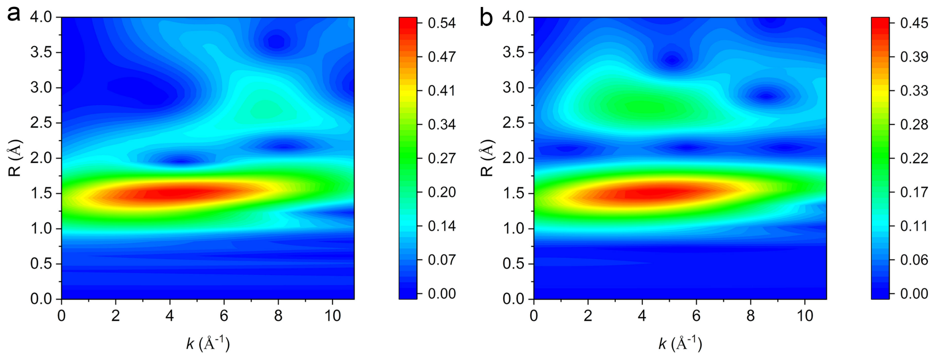

Supplementary Fig. 9 | XAFS wavelet transform (WT) for the reference samples. a, Commercial CuPc. b, Commercial CuO. The y-axis of the WT plots shows the radial distance, and the x-axis reflects the *k*-space resolution of the backscattering atom. The coordination environment of Cu in Cu(OH)BTA consisted of Cu-O bond and Cu-N bond according to the CuPc (Cu-N) and CuO (Cu-O) reference.

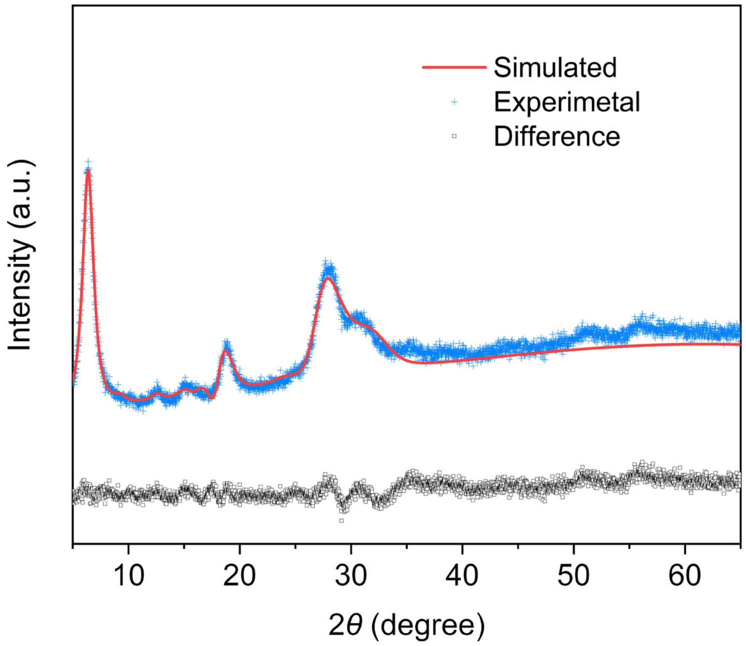

Supplementary Fig. 10 | Experimental (blue) and simulated (red) PXRD patterns of Cu(OH)BTA. The calculated refinement is based on the structure simulated by DFT calculations. The black curve indicates the differences between the experimental data and the refinement. The weighted-profile R factor R_{wp} = 6.59%.

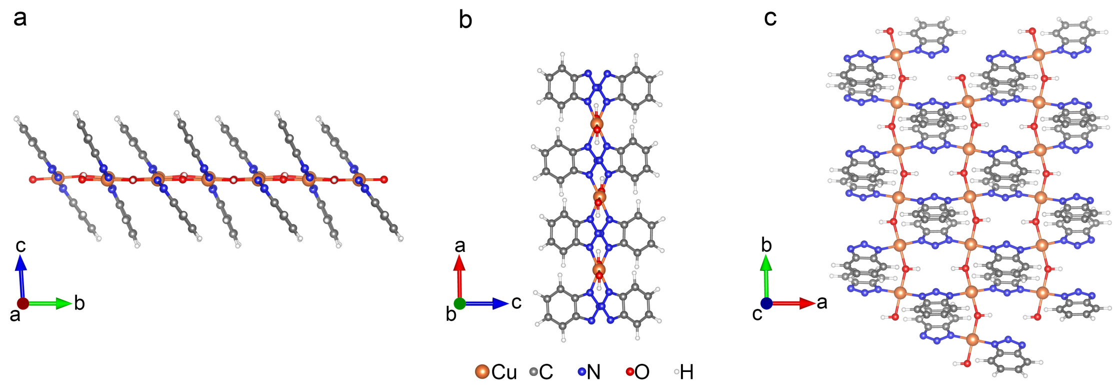

Supplementary Fig. 11 | Model of the Cu(OH)BTA. Views from the a-axis (a), b-axis (b), and c-axis (c). The calculated theoretical mass percentage of elements in Cu(OH)BTA (Calc.: Cu: 30.67%, C: 34.79%, N: 20.28%, O: 11.58%, H: 2.68%).

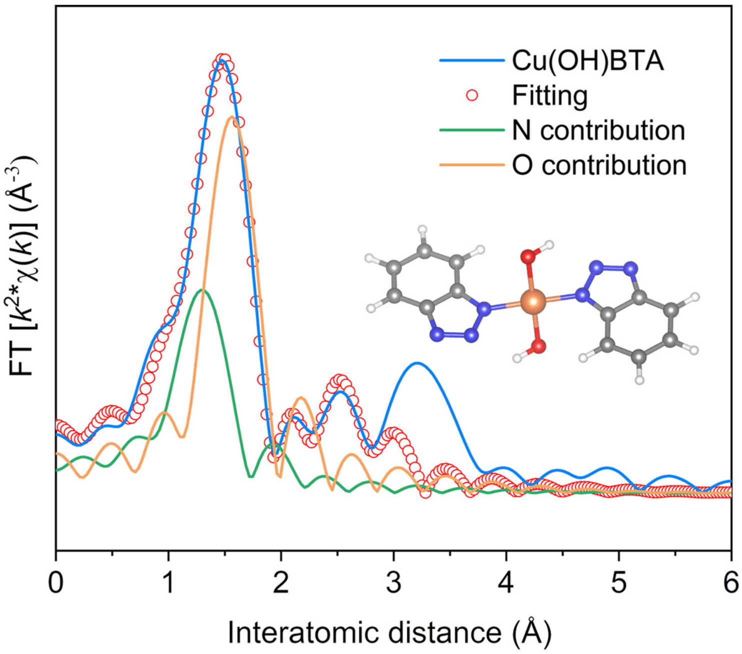

Supplementary Fig. 12 | Cu K-edge EXAFS fitting of Cu(OH)BTA. Inset: an illustration of Cu(OH)BTA local structure near the Cu atom. The orange, gray, blue, red, and white balls represent Cu, C, N, O, and H, respectively.

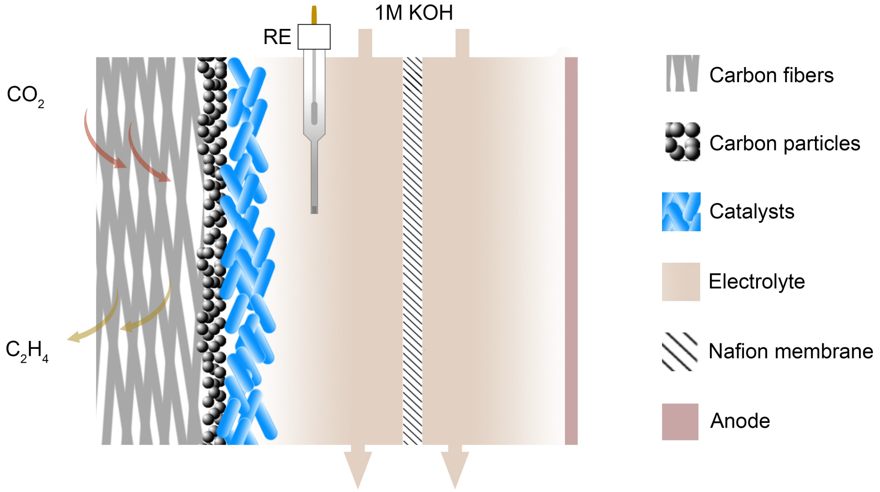

Supplementary Fig. 13 | Schematic illustration of the flow cell used for CO_2RR performance evaluation. The flow cell consists of one gas chamber and two electrolyte chambers. The working electrode consists of gas diffusion layer (GDL, Sigracet 29BC) and catalysts which were sandwiched between the gas chamber and catholyte chamber. 29BC had a hierarchical structure containing a carbon fiber layer and a carbon particle microporous layer. A Ag/AgCl electrode (3 M KCl solution) as a reference electrode was inserted in the catholyte chamber. The reference electrode was as close to the working electrode as possible to minimize the solution resistance. Pt wire was used as the counter electrode. 1 M KOH electrolytes were circulated through the catholyte and anolyte chambers, respectively, using a peristaltic pump. The CO_2 gas flowed into the gas chamber at a constant flow rate controlled by a mass flow meter.

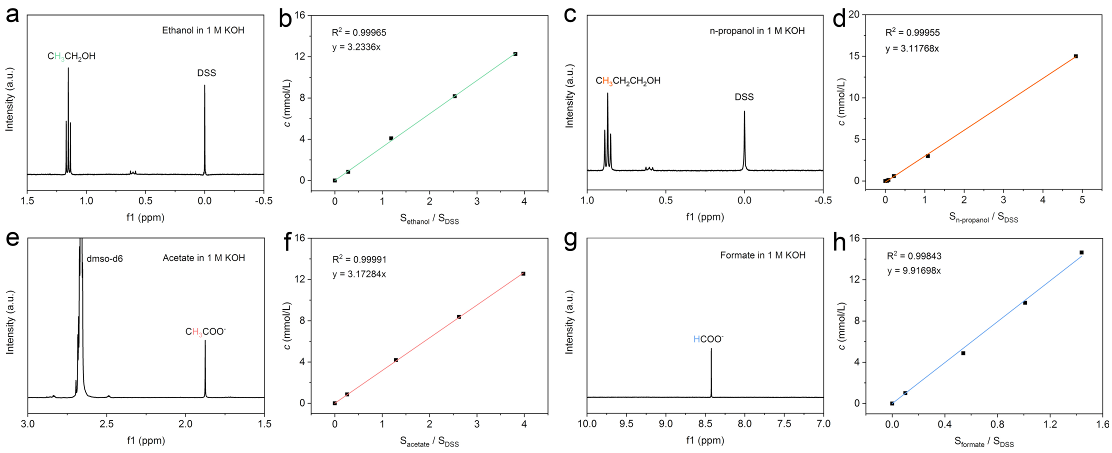

Supplementary Fig. 14 | ^{1}H-NMR spectra of liquid products during CO_2RR and calibration plots. 1H-NMR spectrum (a) and calibration plot (b) for ethanol measured in 1 M KOH electrolyte. 1H-NMR spectrum (c) and calibration plot (d) for n-propanol measured in 1 M KOH electrolyte. 1H-NMR spectrum (e) and calibration plot (f) for acetate measured in 1 M KOH electrolyte. 1H-NMR spectrum (g) and calibration plot (h) for formate measured in 1 M KOH electrolyte. 6 mmol L^{-1} of 3-trimethylsilyl-1-propane sulfonic acid sodium salt (DSS) was used as an internal standard to quantify the concentration of each liquid product.

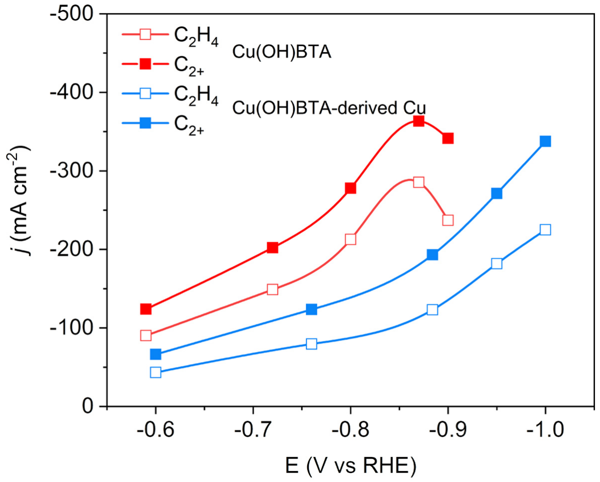

Supplementary Fig. 15 | Partial current densities for C_{2+} and C_2H_4 products over Cu(OH)BTA and Cu(OH)BTA-derived Cu catalysts.

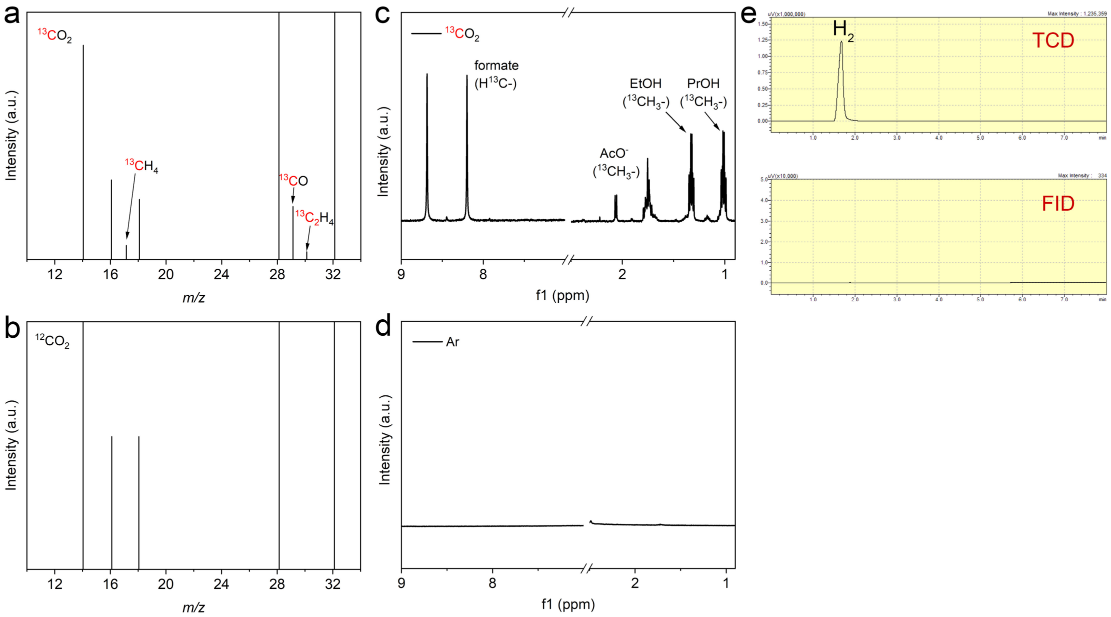

Supplementary Fig. 16 | $^{13}CO_2$ isotope electrochemical experiments over Cu(OH)BTA. a,b, Mass spectra of gas products obtained in $^{13}CO_2$ (a) and $^{12}CO_2$ (b) atmosphere recorded on high-resolution mass spectrometry. c, ^{1}H NMR spectrum of isotope-labeled liquid products. d, ^{1}H NMR spectrum of the liquid product obtained under Ar atmosphere. e, GC profiles of gas product obtained from thermal conductivity detector (TCD) and flame ionization detector (FID) under Ar atmosphere.

The experiment was conducted in 1 M KOH electrolyte. The high-resolution mass spectrometry spectra showed three ^{13}C-labeled gas products, $^{13}C_2H_4$, $^{13}CH_4$, and ^{13}CO at *m/z* = 30, 17, and 29, respectively, when $^{13}CO_2$ was used as the feed. In comparison, these three peaks were absent in the spectrum when $^{12}CO_2$ was used. The peaks of $^{12}C_2H_4$, $^{12}CH_4$, and ^{12}CO at *m/z* = 28, 16, 28 were overlapped by the peak at *m/z* = 28 for N_2, and *m/z* = 16 for O, due to notorious contamination of air during gaseous species analysis (*m/z* = 32 for O_2 for the same reason). To further confirm the source of carbon in the products, we characterized the liquid products using ^{1}H NMR. The spectrum of catholyte revealed the spin-splitting signals of formate, acetate, ethanol, and n-propanol under $^{13}CO_2$ atmosphere, suggesting that $^{13}CO_2$ serves as the only carbon source for liquid products. In addition, no carbonaceous product was detected when we conducted electrocatalysis under Ar atmosphere. Collectively, the carbon in the products originated from the CO_2 gas rather decomposition of the catalysts.

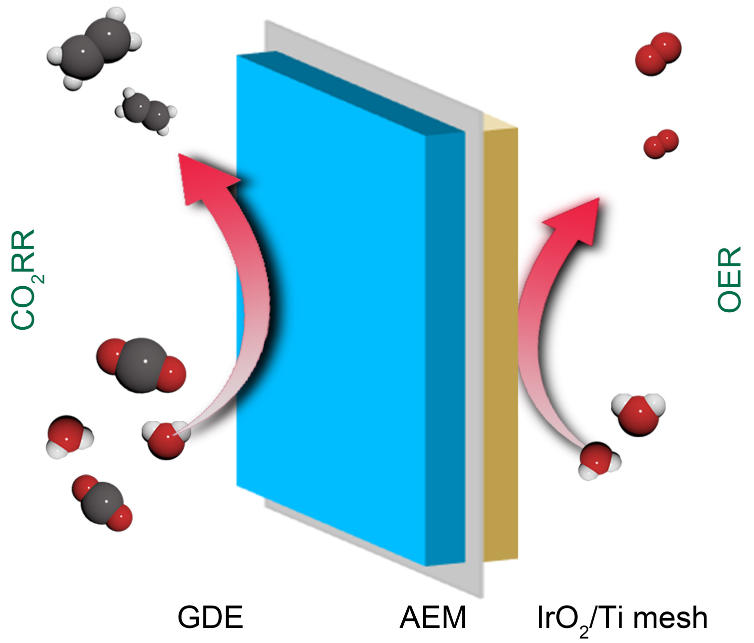

Supplementary Fig. 17 | Schematic illustration of the MEA system. GDE, AEM, and IrO_2/Ti mesh formed a sandwich structure.

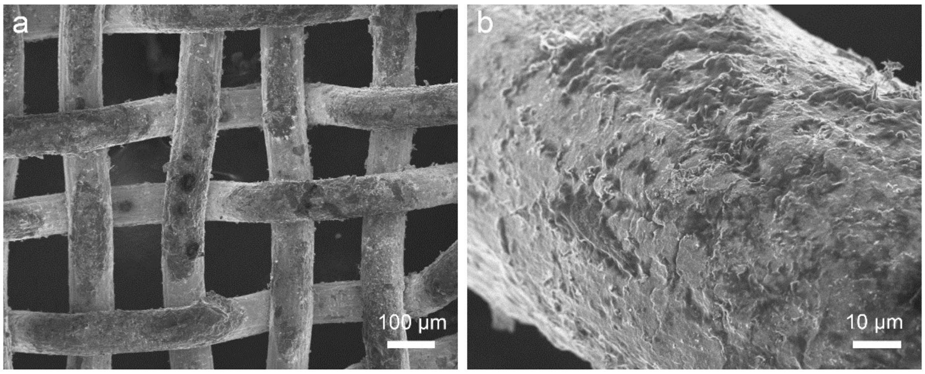

Supplementary Fig. 18 | Morphology of IrO_2/Ti mesh electrode used in the MEA system. SEM image (a) and zoom-in SEM image (b) of the electrode.

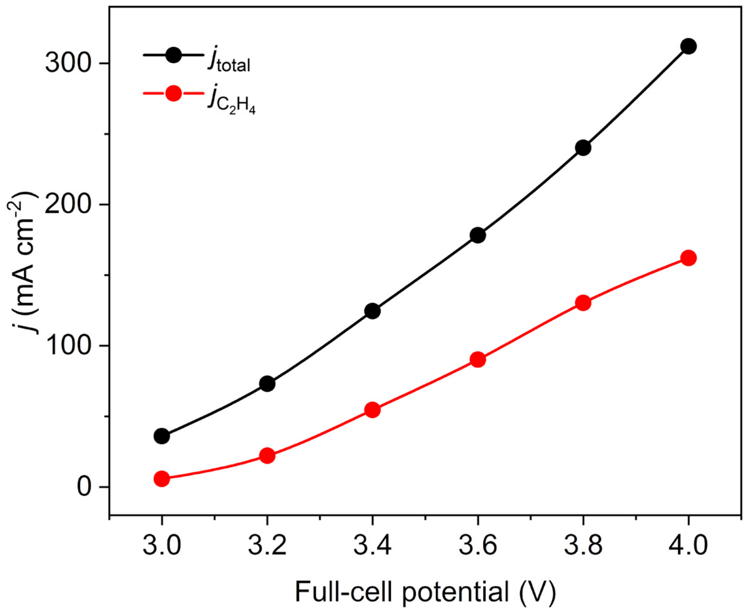

Supplementary Fig. 19 | Total current density and C_2H_4 partial current density over Cu(OH)BTA at different full-cell potentials measured in the MEA system.

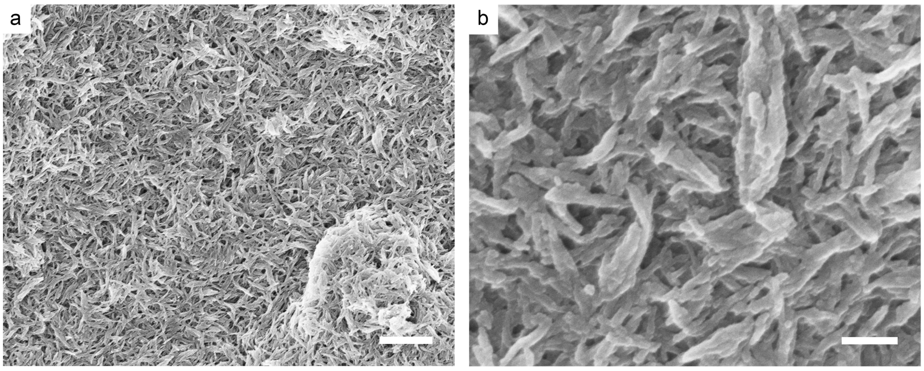

Supplementary Fig. 20 | Scanning electron microscopy (SEM) images of Cu(OH)BTA after stability test in the MEA system. The scale bar is 1 μm in SEM image (a) and 200nm in SEM image (b). The structure of Cu(OH)BTA remained the same after the stability test.

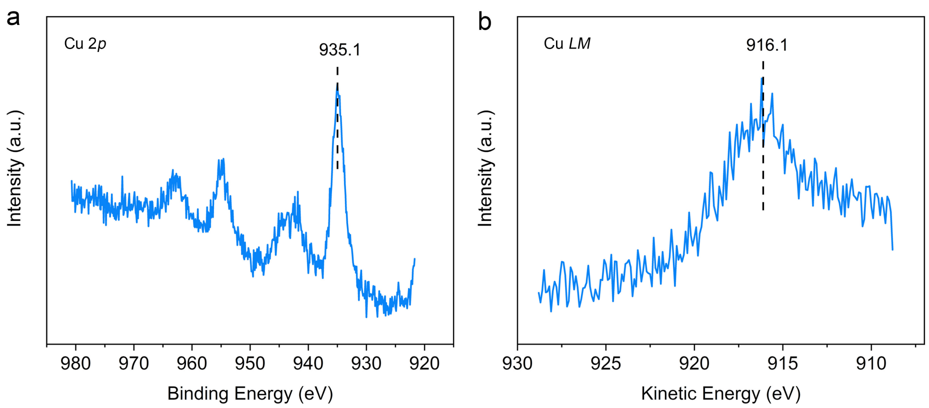

Supplementary Fig. 21 | High-resolution Cu XPS of Cu(OH)BTA after CO_2RR test. a, Cu 2*p* spectrum. b, Cu *LM* spectrum.

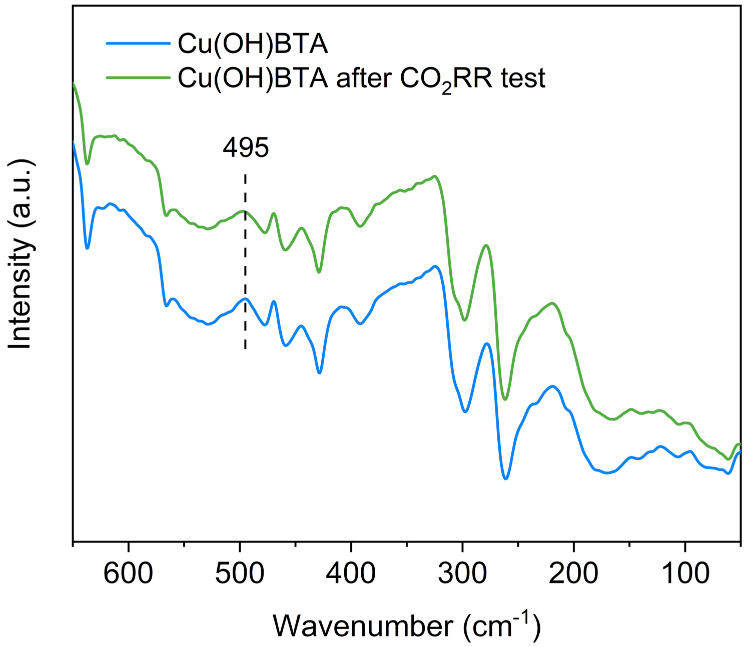

Supplementary Fig. 22 | Synchrotron terahertz far-infrared (THz-far IR) spectra of Cu(OH)BTA before and after CO_2RR test. The Cu-N bond located at 495 cm^{-1} remained which suggested that the coordination structure of Cu(OH)BTA remained after the CO_2RR test.[4]

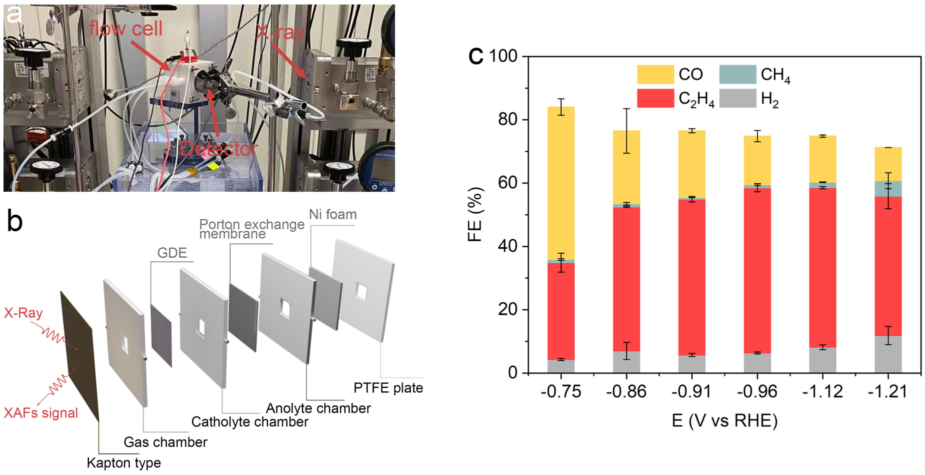

Supplementary Fig. 23 | a, The photograph of operando XAFS experiments in operation. b, The detailed schematic of the operando flow-cell electrolyzer. c, The CO_2RR performance of Cu(OH)BTA performed in the operando flow-cell electrolyzer.

The operando XAS experiments were conducted in a home-made flow cell. Its only difference with the flow cell used to evaluate CO_2RR performance is that we used the Kapton tape to seal the gas chamber to allow for X-ray irradiation in and fluorescent signal out. While analyzing gas products at synchrotron radiation site is not practical, we instead evaluated CO_2RR performance in our lab using the same operando XAS flow cell and at the same experimental conditions. The FEs for each gas product showed almost the same values and current densities at each applied potential, in comparison with those in the normal flow cell. The loading of the catalysts and the GDL used for operando XAS were the same as those in catalytic activity measurements. In sum, we believe that the XAS results in the manuscript presented the active state of the working catalyst.

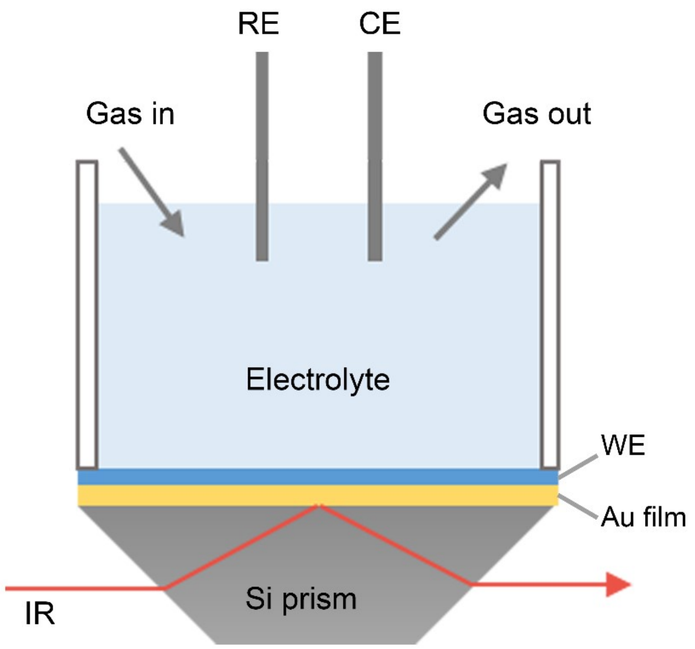

Supplementary Fig. 24 | Schematic illustration of *in-situ* FTIR cell with internal reflection configuration.

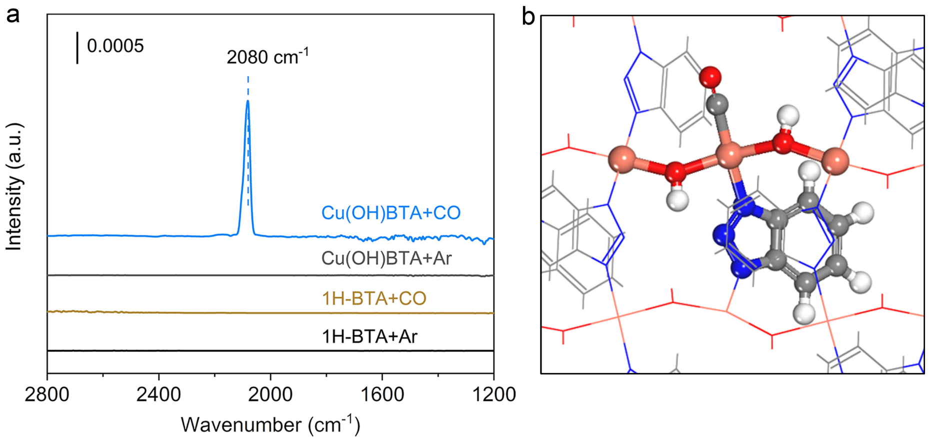

Supplementary Fig. 25 | *CO$_{atop}$ configuration on Cu(OH)BTA. a, DRIFT spectra of Cu(OH)BTA and 1H-BTA molecule after Ar or CO treatment for 30 min. b, The simulated *CO$_{atop}$ configuration on Cu(OH)BTA by DFT calculations. The local structure is highlighted with the ball-and-stick model. The orange, red, blue, grey and white balls represent Cu, O, N, C and H, respectively. The band at 2080 cm^{-1} in DRIFT spectroscopy was assigned to the *CO$_{atop}$ on Cu(OH)BTA, which agreed well with the theoretical band at 2086 cm^{-1} based on the simulated *CO$_{atop}$ configuration by DFT calculations.

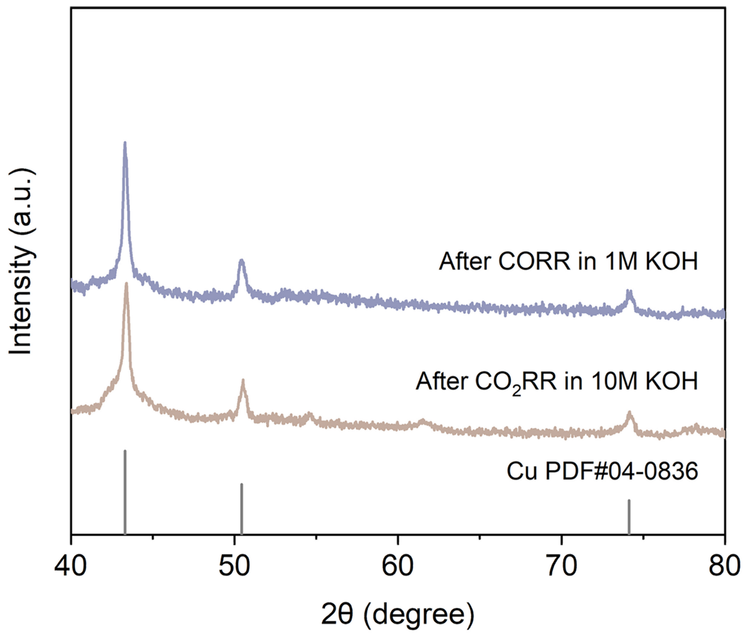

Supplementary Fig. 26 | The XRD patterns of Cu(OH)BTA after CORR measurement in 1 M KOH electrolyte and CO_2RR measurement in 10 M KOH electrolyte, respectively.

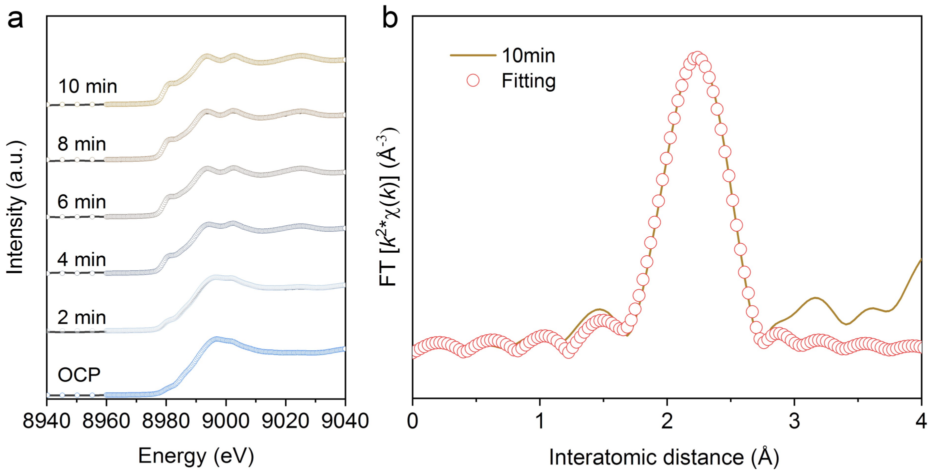

Supplementary Fig. 27 | *in-situ* XAFS measurements. Cu K-edge XANES of Cu(OH)BTA catalysts at a constant current density of -200 mA cm^{-2} under Ar atmosphere. a, Cu K-edge XANES spectra at a time intervals of 2 min. b, The Cu K-edge EXAFS fitting spectrum of as-reduced Cu(OH)BTA after electroreduction for 10 min. Cu(OH)BTA was reduced to metallic Cu.

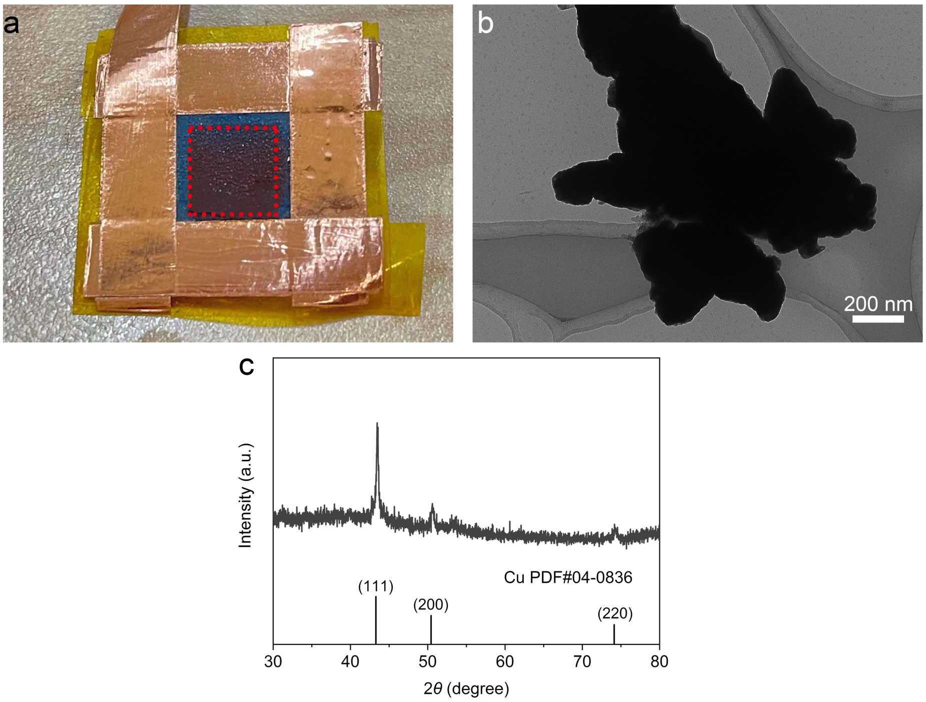

Supplementary Fig. 28 | Characterization of Cu(OH)BTA after electroreduction under Ar atmosphere. a, The photograph of the gas diffusion electrode after electroreduction at -200 mA cm^{-2} for 10 min. b, Transmission electron microscopy (TEM) image of the rectangular area in a. c, The XRD patterns of the rectangular area in a. The Cu standard pattern (PDF#04-0836) was used as the reference. The phase of metallic Cu was formatted quickly under Ar atmosphere, which was consistent with the result of *in-situ* XAFS.

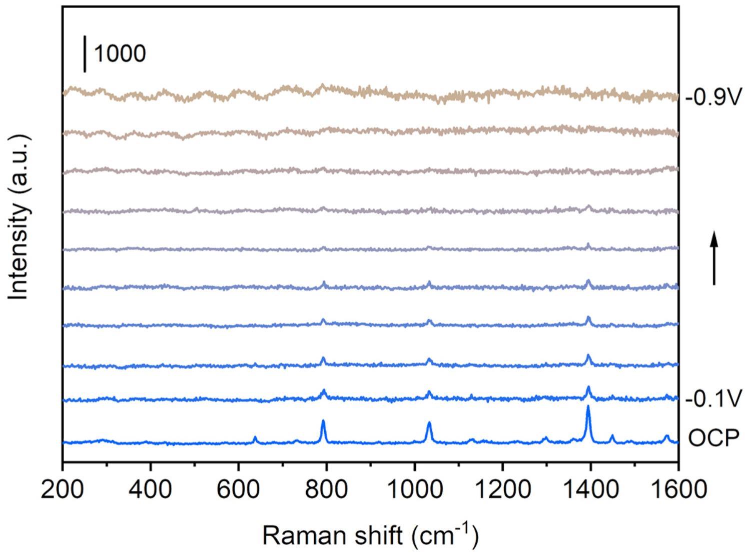

Supplementary Fig. 29 | *in-situ* Raman spectra of Cu(OH)BTA at the applied potential of -0.1 V to -0.9 V under Ar atmosphere. We conducted in-situ Raman spectroscopy of Cu(OH)BTA at an applied potential of -0.1 V to -0.9 V under Ar atmosphere and found that no residual BTA molecule, staying on or coordinating with the surface of the as-reduced Cu nanoparticle, was observed within the detection limit of Raman spectroscopy. In detail, at the open-circuit potential (OCP), Cu(OH)BTA exhibited typical Raman shifts that correspond to the vibrational modes of Cu(OH)BTA; The intensity of the Raman shifts decreased and completely disappeared at potentials lower negative than -0.6 V.

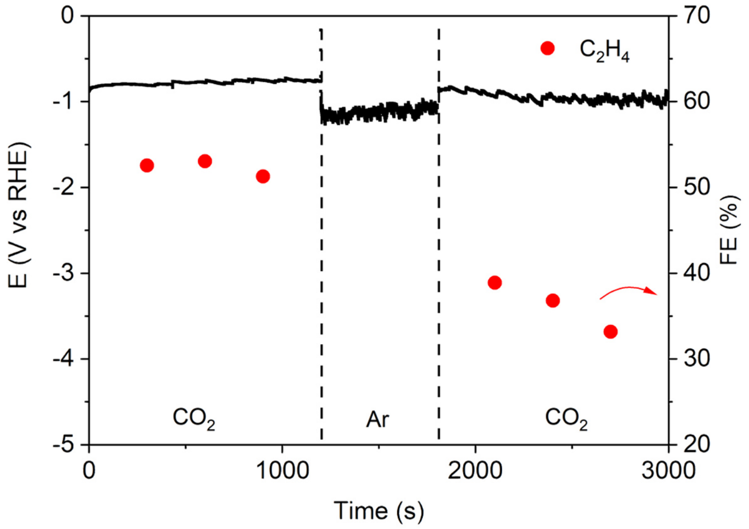

Supplementary Fig. 30 | The trend of CO_2RR performance at -400 mA cm^{-2} over Cu(OH)BTA under different atmosphere. To prove the repeatability of the behavior of Cu(OH)BTA under CO_2RR and Ar conditions, we have examined the CO_2RR gas products before and after Ar atmosphere. At a constant applied current of -400 mA cm^{-2}, the ethylene FE dropped from around 52% to about 36% after CO_2 was replaced by Ar for 10 minutes.

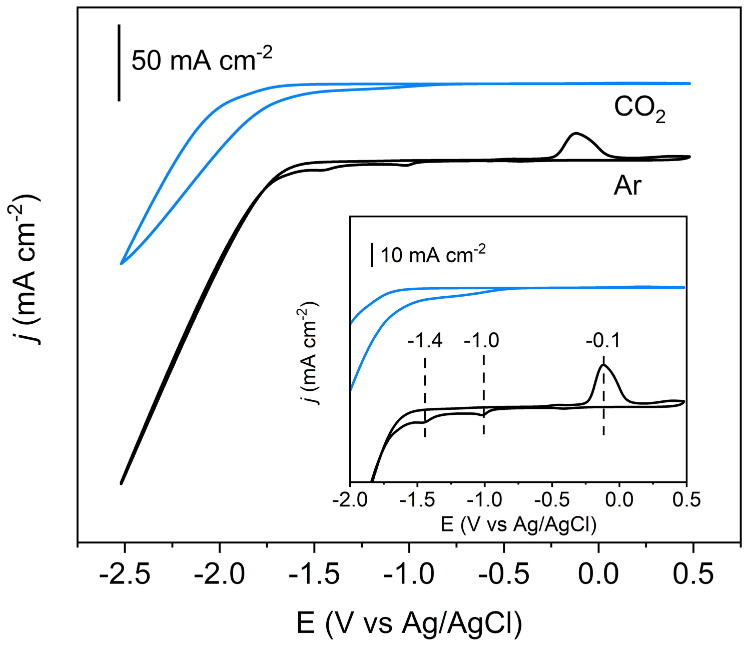

Supplementary Fig. 31 | Cyclic voltammetry (CV) curves of the Cu(OH)BTA electrode in 1 M KOH electrolyte at a scan rate of 100 mV s^{-1} from -2.5 V to 0.5 V (vs Ag/AgCl) under CO_2 and Ar atmosphere, respectively. Inset: the zoom-in CV curves. Obvious reduction peak at -1.0 V (vs. Ag/AgCl) (Cu^{2+} to Cu^{1+}) and -1.4 V (vs. Ag/AgCl) (Cu^{2+} to Cu^0 and Cu^{1+} to Cu^0), and oxidation peak at -0.1 V (vs. Ag/AgCl) (Cu^0 to Cu^{2+}) appeared under Ar atmosphere.[5,6]

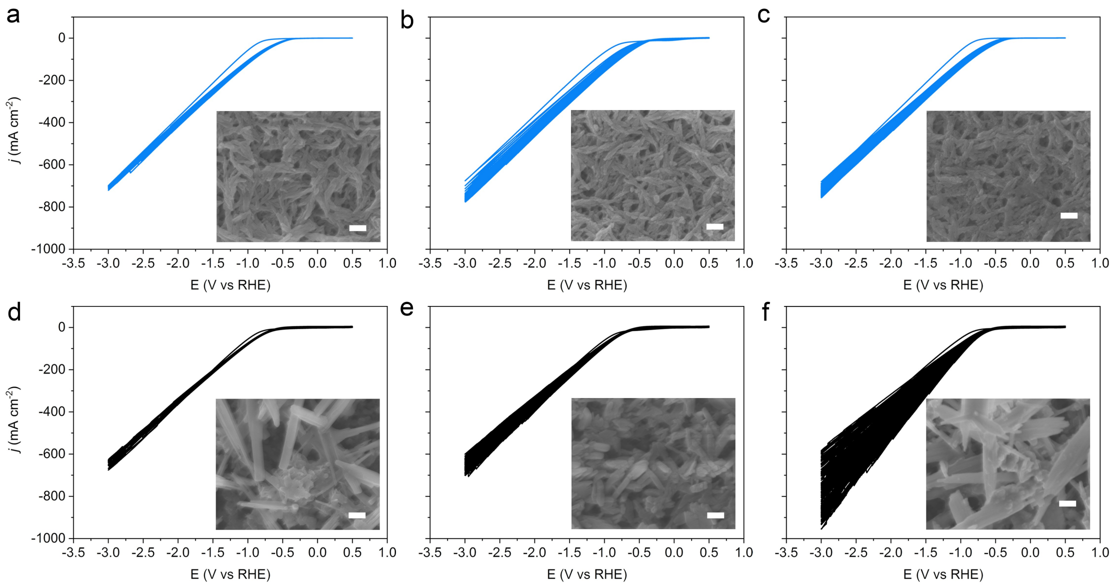

Supplementary Fig. 32 | The CV curves of Cu(OH)BTA collected at different scans under CO_2 and Ar atmosphere. (a) 10 scans. (b) 50 scans. (c) 100 scans under CO_2 atmosphere. (d) 10 scans. (e) 50 scans. (f) 100 scans under Ar atmosphere. Insert: the corresponding SEM images after CV treatment. The scan rate was 100 mV/s. The bar in SEM images is 200 nm.

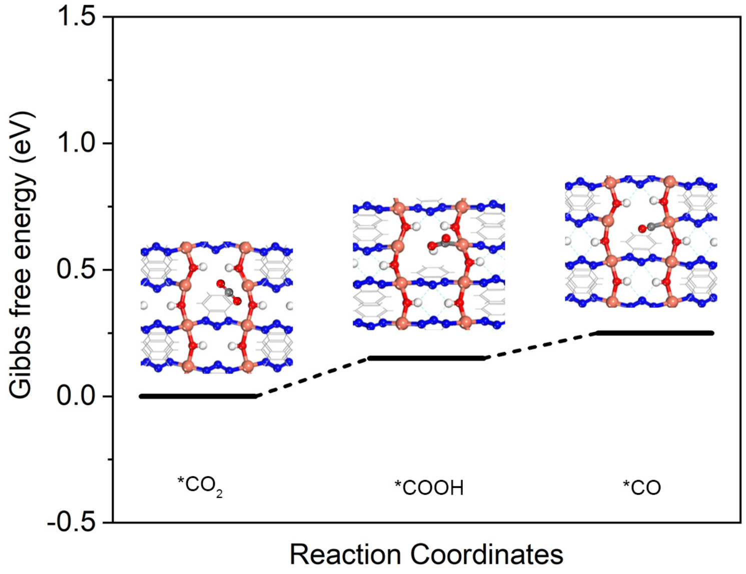

Supplementary Fig. 33 | Gibbs free energy diagram of CO_2RR from $*CO_2$ to *CO pathway on Cu(OH)BTA slab.

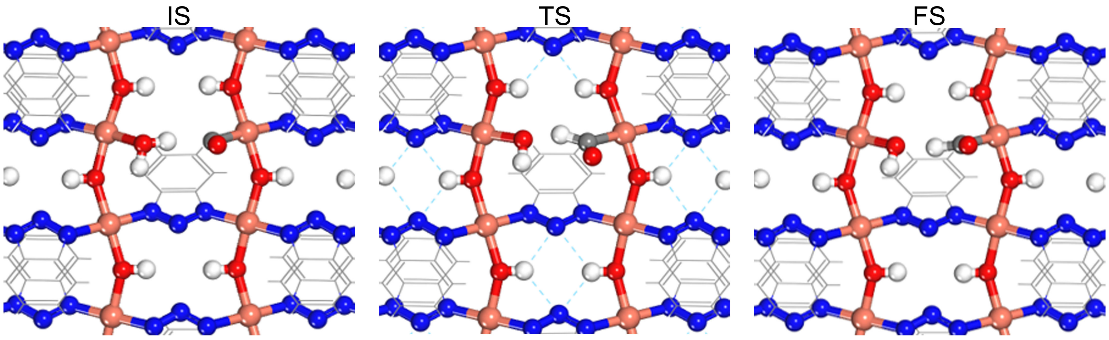

Supplementary Fig. 34 | Geometries of initial (IS), transition (TS), and final state (FS) of hydrogenation of *CO on Cu(OH)BTA slab. Top views of IS (*H_2O + *CO), TS, and FS (*OH + *CHO). The structure of the top layer of Cu(OH)BTA slab was illustrated by ball-and-stick model and the structure of the lower layer was simplified to the wireframe model. This notation is used throughout the Supplementary Information.

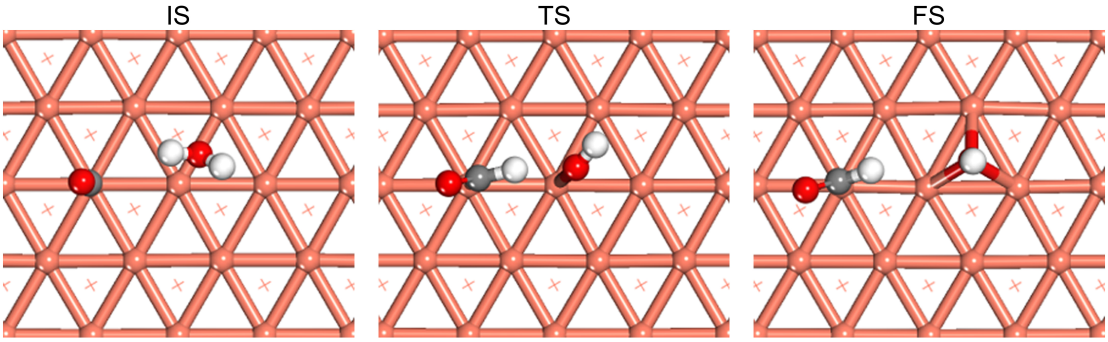

Supplementary Fig. 35 | Geometries of IS, TS, and FS of hydrogenation of *CO on Cu(111) slab. Top views of IS (*H_2O + *CO), TS, and FS (*OH + *CHO).

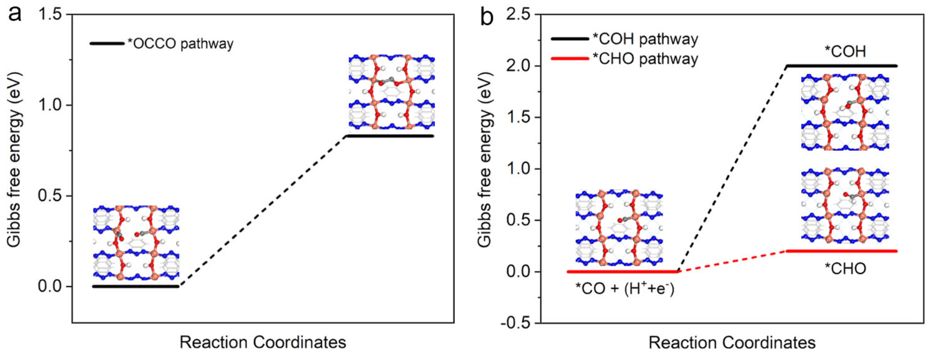

Supplementary Fig. 36 | a, Gibbs free energy diagram of coupling of two *CO to *OCCO on Cu(OH)BTA. Inset figures: top-view geometries of corresponding intermediates (*CO, and *OCCO). b, Gibbs free energy diagram of hydrogenation of *CO to *CHO or *COH on Cu(OH)BTA. Inset figures: top-view geometries of corresponding intermediates (*CO, *COH, and *COH).

We have done further theoretical studies to compare energies of possible reaction pathways for C-C coupling, namely CO–CO, CO–CHO, and CO–COH, three most widely accepted paths in prior reports[7-9]. Our DFT calculation results show a reaction energy for CO–CO coupling of 0.80 eV, much higher than that for OC–CHO coupling (0.14 eV). Due to a prohibitively high energy barrier for the formation of *COH from *CO hydrogenation, the CO–COH path is very unlikely.

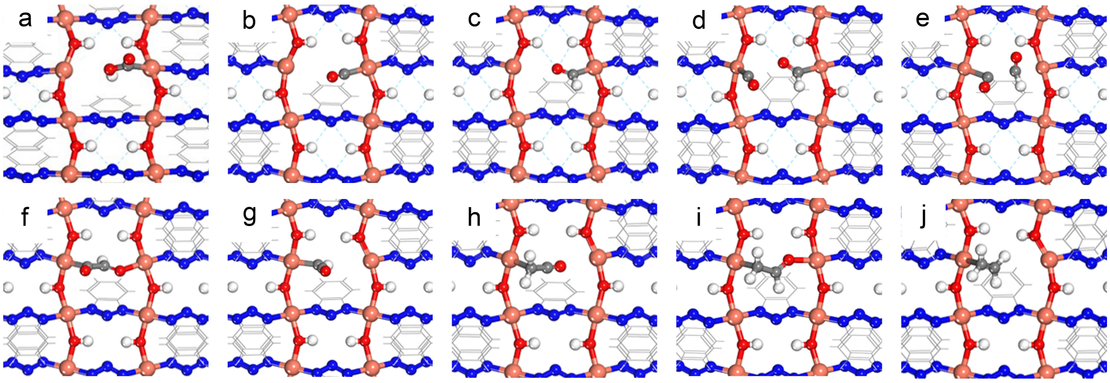

Supplementary Fig. 37 | The evolution of main intermediates from *COOH to *CH_2CH_2 over Cu(OH)BTA during the CO_2 to C_2H_4 process. Top views of (a) *COOH; (b) *CO (c) *CHO; (d) *CHO + *CO; (e) transition state of *OCCHO; (f) *OCCHO; (g) *CHCO; (h) *CH_2CO; (i) *CH_2CHO; (j) *CH_2CH_2 configurations.

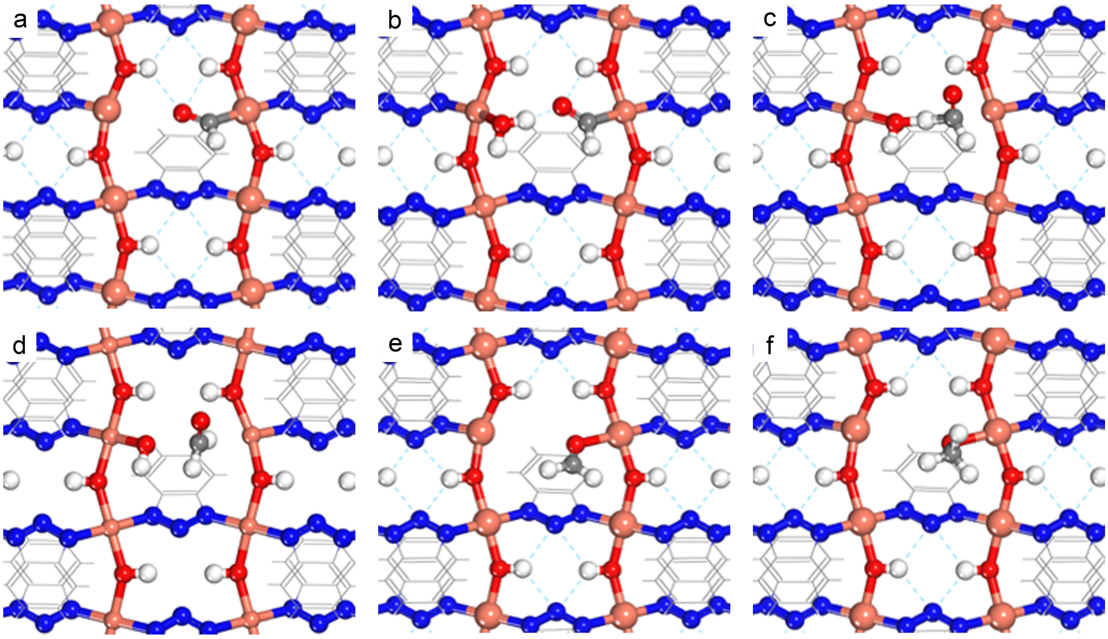

Supplementary Fig. 38 | Geometries of intermediates during CH_4 formation process on Cu(OH)BTA. Top views of (a) *CHO; (b) *H_2O + *CHO; (c) $TS_{C\text{-}H}$; (d) *OH + CH_2O; (e) *OCH_2; (f) *OCH_3.

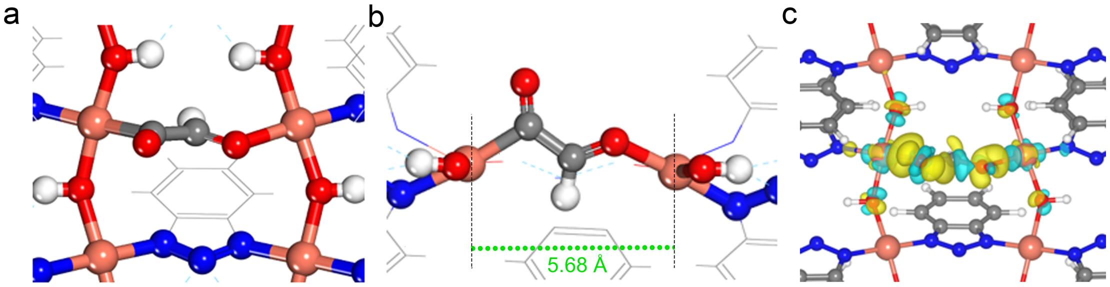

Supplementary Fig. 39 | *OCCHO geometric configuration. Top view (a) and side view (b) of *OCCHO intermediates formed at neighboring Cu sites over Cu(OH)BTA. As shown in b, the optimized length of *OCCHO is 5.68 Å, which matches well with the distance of neighboring Cu sites. c, Top view of charge difference of *OCCHO in Cu(OH)BTA slab. Cyan and yellow contours present electron depletion and electron accumulation, respectively. The iso-surface level is set to be 0.003 eV $Bohr^{-3}$.

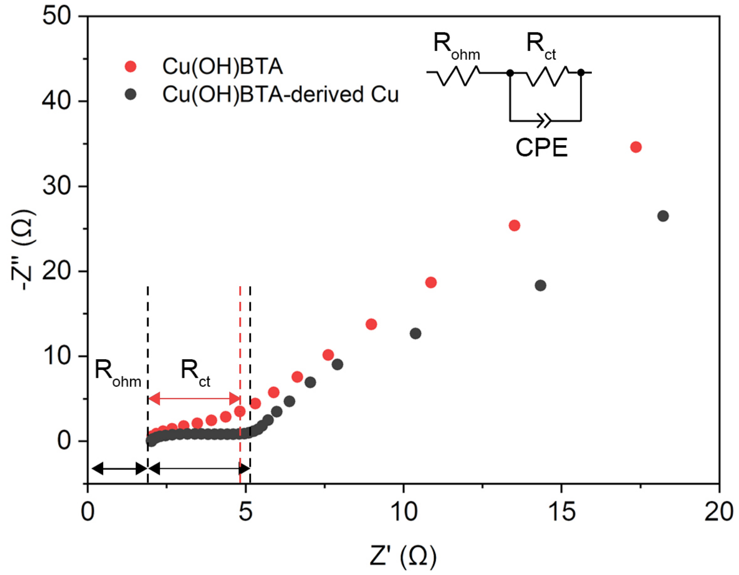

Supplementary Fig. 40 | Electrochemical impedance spectroscopy (EIS) of Cu(OH)BTA and Cu(OH)BTA-derived Cu. The inset equivalent circuit is used for modeling the measured electrochemical response. R_{ohm} represents the solution resistance; R_{ct} represents the interfacial charge transfer resistance. CPE represents double-layer capacitance. The R_{ohm} of Cu(OH)BTA and Cu(OH)BTA-derived Cu is 2.00 ± 0.02 Ω. The R_{ct} of Cu(OH)BTA and Cu(OH)BTA-derived Cu is 2.80 ± 0.10 Ω and 3.10 ± 0.15 Ω, respectively. The Cu(OH)BTA has a fast charge transfer rate as the derived Cu, so the catalysts have such high current densities. The error bars for R_{ohm} and R_{ct} uncertainty represent one standard deviation based on three independent samples.

Supplementary Table 1 | The atomic ratio of N, Cu in Cu(OH)BTA. The result was determined by STEM-EDS mapping.

Catalyst	N (At. %)[a]	Cu (At. %)[a]
Cu(OH)BTA	19.79	6.31

[a] atomic percentage

Supplementary Table 2 | The average atomic ratio of N, Cu elements in Cu(OH)BTA. The results were determined by three independent XPS measurements. The average ratio of N and Cu is 2.75 ± 0.03.

Cu(OH)BTA	N 1*s* (At. %)[a]	Cu 2*p* (At. %)[a]
average	23.96	8.72

[a]atomic percentage.

Supplementary Table 3 | EXAFS fitting data at the Cu K-edge of Cu(OH)BTA.

Catalyst	Scatter path	CN_{EXAFS} /CN_{DFT}	R_{EXAFS}/R_{DFT} (Å)	ΔE_0 (eV)	σ^2 (Å^2)
Cu(OH)BTA	Cu-N	2±0.4 / 2	1.81±0.09 / 1.91	7.7±2.3	0.012±0.001
	Cu-O	2.3±0.3 / 2	1.97±0.02 / 1.96	9.4±1.2	0.018±0.002

CN_{DFT}: coordination numbers calculated by DFT. R_{DFT}: bond distance calculated by DFT. CN_{EXAFS}: coordination numbers simulated by EXAFS fitting. R_{EXAFS}: bond distance simulated by EXAFS fitting. σ^2: the Debye-Waller factor. ΔE_0: the inner potential shift.

Supplementary Table 4 | Element analysis of Cu(OH)BTA. The error bars for the data uncertainty represent one standard deviation based on three independent samples.

**Elements**	**Calc.**[a]	**Found**[a]
**C (%)**	34.79	35.70 ± 0.07
**N (%)**	20.28	20.36 ± 0.62
**H (%)**	2.68	2.71 ± 0.09

[a]mass percentage.

Supplementary Table 5 | Summary of the amount of Cu in Cu(OH)BTA obtained by ICP-AES. The error bars for the data uncertainty represent one standard deviation based on three independent samples.

Cu(OH)BTA	Cu (wt. %)[a]
**1**	31.38
**2**	29.32
**3**	31.53
**average**	30.74 ± 1.24

[a]mass percentage.

Supplementary Table 6 | Comparison of C_2H_4 half-cell efficiency over Cu(OH)BTA and Cu(OH)BTA-derived Cu under different applied potential.

catalysts	Applied potential (vs. RHE)	Energy efficiency (%)
Cu(OH)BTA	-0.59	28.5
	-0.72	29.2
	-0.80	30.1
	-0.87	31.3
	-0.90	21.3
Cu(OH)BTA-derived Cu	-0.60	13.6
	-0.76	15.3
	-0.88	16.7
	-0.95	19.2
	-1.00	19.3

Supplementary Table 7 | Comparison of the maximum C_{2+}, C_2H_4 faradaic efficiencies, current density, and durability over Cu(OH)BTA and the start-of-the-art Cu-based catalysts that were derived from molecular compounds. NA: not available.

Catalysts	FE_{C2+} (%)	FE_{C2H4} (%)	Current density (mA cm^{-2})	Durability (h)	Ref.
Cu(OH)BTA	73	57.1	500	67	**This work**
HKUST-1	~69	45	262	~2.7	10
PorCu	~22	17	49	1.2	11
BIF-102NSs	NA	11.3	14	NA	12
OD-Cu-III	74.9	~43	300	50	13
Cu-PzH	NA	60	~577	~4	14
Cu3-Br	NA	55	300	9.5	15
PcCu-Cu-O	NA	50	7.3	4	16
Cu-CeO_2-4%	~20	15	70	2.2	17
Cu-N-C-800	NA	24.8	15.4	10	18
Cutrz	80	50	280	35	19

Supplementary Table 8 | Peak assignments of Cu(OH)BTA in Raman spectroscopy.[20]

Raman shift (cm^{-1})	Assignments
639	Triazole ring torsion
793	Benzene ring breathing
1035	In-plane triagonal ring breathing
1395	Triazole ring stretch
1574	Phenyl ring stretch

Supplementary Table 9 | Linear combination fitting (LCF) of *in-situ* XAFS of Cu(OH)BTA under Ar atmosphere.

LCF-XAFS	The ratio of Cu(OH)BTA (%)	The ratio of Cu (0) (%)	[a]CN	[b]R (Å)
2 min	59	41	-	-
4 min	22	78	-	-
6 min	0	100	10.2	2.54
8 min	0	100	10.5	2.54
10 min	0	100	10.6	2.54

[a]CN: coordination numbers. [b]R: bond distance.

Supplementary Table 10 | The distance of neighboring Cu sites over Cu(OH)BTA during the C-C coupling step.

Adsorbed intermediates	The distance of neighboring Cu (Å)
slab	5.70
*CO	5.77
*CHO	5.83
*OCCHO	5.68

Supplementary Table 11 | The zero-point energy corrections of key gas species during the CO_2RR process.

Species	Free energy (eV)	ZPE corrections (eV)
CO_2(g)	-22.98	0.31
H_2(g)	-6.76	0.27
CO(g)	-14.79	0.14
H_2O(g)	-14.22	0.57
C_2H_4(g)	-31.96	1.36
CH_4(g)	-24.03	1.20
BTA	-96.75	2.82

Supplementary References

1. Ma, H. *et al.* Study of cyano and hydroxyl groups modification on the properties of porous carbon nitride synthesized by using a salt assistant method. *Appl. Surf. Sci.* **507**, 144885 (2020).
2. Zhong, S. *et al.* Efficient electrochemical transformation of CO_2 to C_2/C_3 chemicals on benzimidazole-functionalized copper surfaces. *Chem. Commun.* **54**, 11324-11327 (2018).
3. Jagerovic, N., Jimeno, M. a. L., Alkorta, I., Elguero, J. & Claramunt, R. M. a. An experimental (NMR) and theoretical (GIAO) study of the tautomerism of benzotriazole in solution. *Tetrahedron* **58**, 9089-9094 (2002).
4. Sithole, R. K. *et al.* Synthesis and characterization of Cu_3N nanoparticles using pyrrole-2-carbaldpropyliminato Cu(II) complex and $Cu(NO_3)_2$ as single-source precursors: the search for an ideal precursor. *New J. Chem.* **42**, 3042-3049 (2018).
5. Wan, Y., Zhang, Y., Wang, X. & Wang, Q. Electrochemical formation and reduction of copper oxide nanostructures in alkaline media. *Electrochem. Commun.* **36**, 99-102 (2013).
6. Deng, Y., Handoko, A. D., Du, Y., Xi, S. & Yeo, B. S. In Situ Raman Spectroscopy of Copper and Copper Oxide Surfaces during Electrochemical Oxygen Evolution Reaction: Identification of Cu^{III} Oxides as Catalytically Active Species. *ACS Catal.* **6**, 2473-2481 (2016).
7. Sha, Y. *et al.* Anchoring Ionic Liquid in Copper Electrocatalyst for Improving CO_2 Conversion to Ethylene. *Angew. Chem. Int. Ed.* **134**, e202200039 (2022).
8. Zhu, H.-L. *et al.* A Porous π–π Stacking Framework with Dicopper(I) Sites and Adjacent Proton Relays for Electroreduction of CO_2 to C_{2+} Products. *J. Am. Chem. Soc.* **144**, 13319-13326 (2022).
9. Zhao, Z.-H., Zhu, H.-L., Huang, J.-R., Liao, P.-Q. & Chen, X.-M. Polydopamine Coating of a Metal-Organic Framework with Bi-Copper Sites for Highly Selective Electroreduction of CO_2 to C_{2+} Products. *ACS Catal.* **12**, 7986-7993 (2022).
10. Nam, D.-H. *et al.* Metal-Organic Frameworks Mediate Cu Coordination for Selective CO_2 Electroreduction. *J. Am. Chem. Soc.* **140**, 11378-11386 (2018).
11. Weng, Z. *et al.* Electrochemical CO_2 Reduction to Hydrocarbons on a Heterogeneous Molecular Cu Catalyst in Aqueous Solution. *J. Am. Chem. Soc.* **138**, 8076-8079 (2016).
12. Shao, P. *et al.* Synthesis of a Boron–Imidazolate Framework Nanosheet with Dimer Copper Units for CO_2 Electroreduction to Ethylene. *Angew. Chem. Int. Ed.* **60**, 16687-16692 (2021).
13. Wu, Z.-Z. *et al.* Identification of Cu(100)/Cu(111) Interfaces as Superior Active Sites for CO Dimerization During CO_2 Electroreduction. *J. Am. Chem. Soc.* **144**, 259-269 (2022).
14. Wang, R. *et al.* Partial Coordination-Perturbed Bi-Copper Sites for Selective Electroreduction of CO_2 to Hydrocarbons. *Angew. Chem. Int. Ed.* **60**, 19829-19835 (2021).
15. Lu, Y.-F. *et al.* Predesign of Catalytically Active Sites via Stable Coordination Cluster Model System for Electroreduction of CO_2 to Ethylene. *Angew. Chem. Int. Ed.* **60**, 26210-26217 (2021).
16. Qiu, X.-F., Zhu, H.-L., Huang, J.-R., Liao, P.-Q. & Chen, X.-M. Highly Selective CO_2 Electroreduction to

C_2H_4 Using a Metal-Organic Framework with Dual Active Sites. *J. Am. Chem. Soc.* **143**, 7242-7246 (2021).

17. Wang, Y. *et al.* Single-Atomic Cu with Multiple Oxygen Vacancies on Ceria for Electrocatalytic CO_2 Reduction to CH_4. *ACS Catal.* **8**, 7113-7119 (2018).
18. Guan, A. *et al.* Boosting CO_2 Electroreduction to CH_4 via Tuning Neighboring Single-Copper Sites. *ACS Energy Lett.* **5**, 1044-1053 (2020).
19. Huang, D.-S. *et al.* A Stable and Low-Cost Metal-Azolate Framework with Cyclic Tricopper Active Sites for Highly Selective CO_2 Electroreduction to C_{2+} Products. *ACS Catal.* **12**, 8444-8450 (2022).
20. Thomas, S., Venkateswaran, S., Kapoor, S., D'Cunha, R. & Mukherjee, T. Surface enhanced Raman scattering of benzotriazole: a molecular orientational study. *Spectrochimica Acta* **60**, 25-29 (2004).
